# Retinoic acid-induced expression of *Hnf1b* and *Fzd4* is required for pancreas development in *Xenopus laevis*

**DOI:** 10.1242/dev.161372

**Published:** 2018-06-08

**Authors:** Maja B. Gere-Becker, Claudia Pommerenke, Thomas Lingner, Tomas Pieler

**Affiliations:** 1 Department of Developmental Biochemistry, University of Goettingen, Justus-von-Liebig-Weg 11, 37077 Goettingen, Germany; 2 Leibniz Institute DSMZ-German Collection of Microorganisms and Cell Cultures, Inhoffenstrasse 7B, 38124 Braunschweig, Germany; 3 Genevention GmbH, Rudolf-Wissel-Str. 28, 37079 Goettingen, Germany

**Keywords:** Pancreas, Retinoic acid, Hnf1β, Fzd4

## Abstract

Retinoic acid (RA) is required for pancreas specification in *Xenopus* and other vertebrates. However, the gene network that is directly induced by RA signalling in this context remains to be defined. By RNA sequencing of *in vitro*-generated pancreatic explants, we identified the genes encoding the transcription factor Hnf1β and the Wnt-receptor Fzd4/Fzd4s as direct RA target genes. Functional analyses of *Hnf1b* and *Fzd4*/*Fzd4s* in programmed pancreatic explants and whole embryos revealed their requirement for pancreatic progenitor formation and differentiation. Thus, Hnf1β and Fzd4/Fzd4s appear to be involved in pre-patterning events of the embryonic endoderm that allow pancreas formation in *Xenopus*.

## Abstract (precis)

**Summary:** The direct RA target genes *Hnf1b* and *Fzd4* act as mediators of RA function in the pre-patterning of the dorsal endoderm during *Xenopus* gastrulation, resulting in pancreas specification in this context.

## INTRODUCTION

The molecular mechanisms of vertebrate pancreas development appear to be largely conserved across species. Although the cascade of transcriptional regulatory events leading to the formation of the different specialised exo- and endocrine cells is understood in some detail, the preceding regionalisation of the endoderm that allows for the specification of a common pool of pancreatic precursor cells remains to be defined more precisely (reviewed in Pan and Wright, 2011; Shih et al., 2013). Lineage-tracing experiments making use of gastrula-stage *Xenopus* embryos revealed that cells within the dorsal endoderm contribute to the formation of the pancreatic organ (Chalmers and Slack, 2000). It was further demonstrated that, during this initial phase of organ specification, pancreatic precursor cells form under the influence of retinoic acid (RA) (Chen et al., 2004; Stafford et al., 2004). An essential role of the RA signalling pathway in the context of pancreas development had been discovered previously in the zebrafish (Stafford and Prince, 2002) and was later found to be equally relevant for mammalian pancreas specification (Martín et al., 2005). The concept of a RA gradient within the dorsal endoderm forming during early gastrula stages of *Xenopus* development is further supported by the expression characteristics of the key enzyme for RA biosynthesis, Raldh2 (Aldh1a2 – Xenbase), and of the major RA-degrading enzyme, Cyp26a1, during gastrulation. These two genes exhibit nonoverlapping expression patterns in the dorsal mesoderm, with Raldh2 being expressed immediately adjacent to the pre-pancreatic endoderm (Hollemann et al., 1998; Chen et al., 2001).

Tissue explants have been used to achieve pancreatic gene expression. Asashima and colleagues were the first to demonstrate RA-induced expression of pancreatic marker genes in dorsal lip explants from early gastrula-stage *Xenopus* embryos (Moriya et al., 2000a). The same group also used early embryonic ectodermal explants to promote the formation of pancreatic tissue by treatment with a combination of activin and RA (Moriya et al., 2000b). A similar result was obtained by programming the same type of explant with a cocktail including VegT, Noggin and RA (Chen et al., 2004; Borchers and Pieler, 2010). This same protocol, which was also utilised in the current study, aims to mimic early key regulatory events, given that RA signalling together with inhibition of BMP activity makes it possible to convert ventral endomesodermal explants from gastrula-stage *Xenopus* embryos to a pancreatic fate (Pan et al., 2007). Similarly, experimental protocols for the *in vitro* generation of β cells from embryonic stem cells (ESCs) (Kroon et al., 2008; Rezania et al., 2012; Schulz et al., 2012; Pagliuca et al., 2014) or induced pluripotent stem cells (iPSCs) (Zhang et al., 2009; Schiesser et al., 2014; Shaer et al., 2015) all include application of RA.

To the best of our knowledge, the RA-dependent gene network that drives pancreatic fate during gastrula stages of embryogenesis remains to be defined. The identification of such RA-dependent regulators of pancreas development could further improve protocols for the *in vitro* generation of pancreatic tissue. With the aim of identifying RA-regulated genes involved in pancreas specification, we made use of the *Xenopus* ectodermal explant system and RNA-sequencing (RNA-seq) analyses resulting in the identification of two direct, endodermally expressed RA target genes, namely *Hnf1b* and *Fzd4*. Functional studies performed *in vitro* and *in vivo* revealed their requirement for pancreas development.

## RESULTS

### Programmed pancreatic explants recapitulate the *in vivo* temporal pattern of pancreatic gene expression

**Fig. 1.**
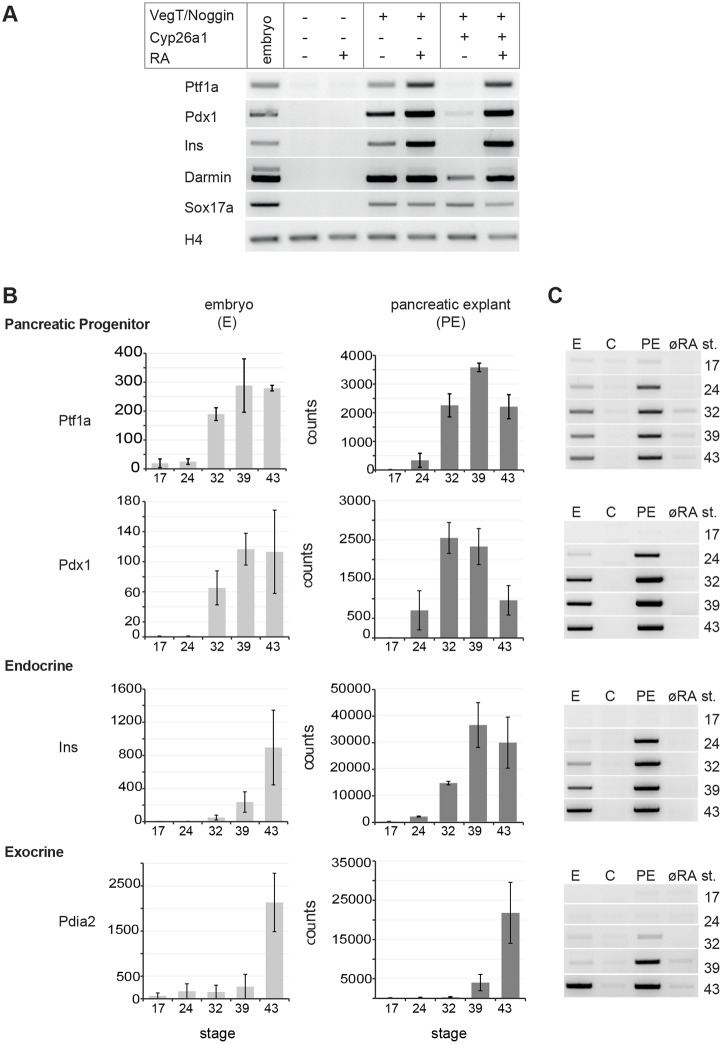
**Comparative analysis of the temporal profile of pancreatic marker gene expression *in vivo* and in programmed explants.** (A) Ectodermal explants isolated from stage 8/9 embryos and injected as indicated were treated with 5 µM RA and cultivated until the equivalent of stage 30. Detection of marker gene expression for the genes indicated was done by RT-PCR. (B) and (C) Transcript quantification using Nanostring analysis (B) and RT-PCR (C) of whole embryos (five embryos per condition) and explants (∼50 explants per condition) from two biological replicates with untreated and treated embryos grown to the equivalent of the developmental stages indicated. Average values are given as mean and error bars as s.e.m. C, unprogrammed explants; E, embryo; Ins, Insulin; øRA, programmed explants with blocked endogenous RA signalling (VegT/Noggin/Cyp26a1); PE, pancreatic explants (VegT/Noggin/RA).

We previously demonstrated that programming of ectodermal explants from *Xenopus* blastulae with a cocktail containing Noggin, VegT and RA is sufficient to drive pancreatic gene expression (Chen et al., 2004). Furthermore, we also found that Noggin by itself induces significant expression levels of the RA-generating enzyme Raldh2 in this system (Pan et al., 2007). Thus, even in the absence of exogenously added RA, a basal level of pancreatic marker gene expression is induced by a combination of VegT and Noggin alone, which appears to be further increased by addition of excess RA (Fig. 1A). To create a control situation that is devoid of RA, we co-injected the RA-degrading enzyme Cyp26a1 together with VegT and Noggin, resulting in the almost complete loss of pancreatic gene expression, which was rescued upon addition of excess exogenous RA (Fig. 1A). We used whole-mount *in situ* hybridisation (WMISH) to estimate the proportion of pancreatic tissue that would form in such programmed explants at different concentrations of RA (Fig. S1A,B). Insulin expression was detected in singular, scattered cells, whereas *Ptf1a* and *Pdx1* expression occurred in coherent groups of cells. Whereas an increase in RA concentration from 5 to 15 µM enhanced pancreatic gene expression to some extent, a further advance to 30 µM had no significant additional effect (Fig. S1C). Under all conditions, a significant portion of the cells in the explants was not positive for pancreatic marker gene transcription, probably because they had adopted a mesodermal or ectodermal fate (see below).

We next examined the temporal pattern of pancreatic progenitor and differentiation marker gene transcription in programmed explants. Representative transcripts were analysed at different time points by RT-PCR as well as by Nanostring analysis in explants and whole embryos (Fig. 1B,C, Table S1). Absolute transcript levels for all pancreatic progenitor and differentiation markers increased more than 10-fold in pancreatic explants compared with whole embryos at maximum expression levels. We also observed a simultaneous onset of transcription for the progenitor markers Ptf1a and Pdx1 at stage 24 in RA-treated explants, but not in Noggin/VegT/Cyp26a1-programmed or control explants. Insulin expression occurred at stage 24 in explants, but, similar to Ptf1a and Pdx1, was somewhat delayed in embryos. The exocrine differentiation marker Pdia2, as well as the endocrine marker glucagon (RT-PCR data, not shown), first increased at the equivalent of stage 39 in pancreatic explants, which was slightly earlier than in stage 43 whole embryos, whereas amylase, as an additional exocrine marker gene, was induced at stage 43 in both explants and embryos (Table S1). Finally, *Tm4sf3* (*Tspan8* – Xenbase), a gene specific for the ventral pancreatic Anlage, was also detected in stage 43 RA-treated explants and control embryos. Induction of lung differentiation, as an example of another endodermal organ, reflected by the expression of surfactant protein, was observed in late-stage embryos but never in programmed explants (Table S1). As expected, early transient expression of the general endodermal marker Sox17 was observed in both pancreatic explants and whole embryos at early stages, but not in a RA-dependent manner. Conversely, early explant expression of Darmin, a different general endodermal marker, was strictly dependent on RA signalling and occurred at a level similar to that in the embryo. Significant levels of RA-independent gene transcription of mesodermal and (neuro-) ectodermal genes were detected in the explants (Table S1).

Taken together, these data revealed the partial but efficient conversion of the pluripotent ectodermal explants to a pancreatic fate, in principle following the temporal profile of pancreas organogenesis in the embryo.

### *Hnf1b* and *Fzd4* are early endodermal RA-responsive genes

**Fig. 2.**
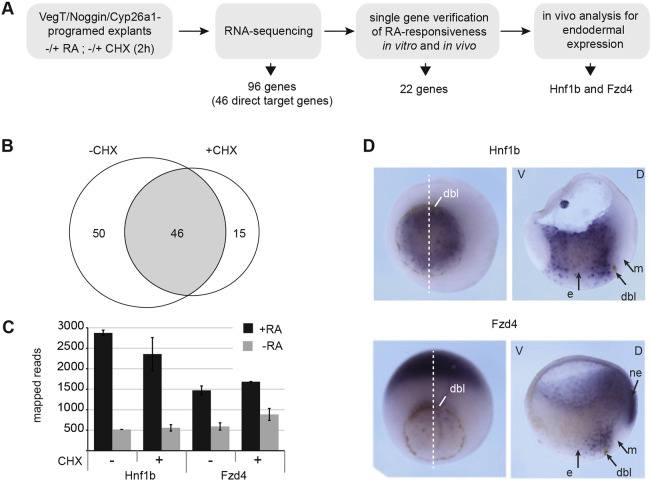
**Identification of *Hnf1b* and *Fzd4* as direct endodermal RA target genes.** (A) Experimental procedure for the identification of early direct RA target genes in the context of pancreas development. (B) Venn diagram comparing genes differentially expressed within 2 h of RA addition in the absence or presence of CHX. A total of 46 putative direct RA target genes were induced under both conditions. (C) RNA-seq results for RA-mediated *Hnf1b* and *Fzd4* induction. The number of mapped reads 2 h after the addition of RA in the presence or absence of CHX is indicated. The data result from two biological replicates with ∼50 explants per condition. Average values are given as mean and error bars as s.e.m. (D) WMISH for *Hnf1b* and *Fzd4* in gastrula-stage embryos. Whole embryos are depicted on the left-hand side and bisected embryos on the right-hand side. dbl, dorsal blastopore lip; e, endoderm; m, mesoderm; ne, neuroectoderm.

In an attempt to identify RA-induced genes that function in pancreas development, we further investigated the pancreatic explant system by using RNA-seq analysis. For the distinction of direct and indirect RA target genes, explants were treated with the translational inhibitor cycloheximide (CHX) (Fig. 2A). It was previously demonstrated that the gene encoding the RA-degrading enzyme, Cyp26a1, which we also found to be induced in the explant system, is a direct RA target gene (Ray et al., 1997; Abu-Abed et al., 1998). *Cyp26a1* was induced in the presence of CHX within 1 h of the addition of RA, and its levels increased further 2 h after treatment initiation (Fig. S9A). Therefore, we performed transcriptome analysis for the identification of other RA target genes under the same conditions. In total, 96 genes were classified as RA targets, 46 of these as direct targets (Fig. 2B, Tables S2 and S3). Using Nanostring analysis, 82 candidate genes from the RNA-seq analysis were tested individually for their RA inducibility in whole embryos as well as in explants. Of these candidate genes, 41 were confirmed in both systems (Fig. S2A,C) and 22 of these were found to be reduced upon inhibition of RA signalling (Fig. S2B,C, Table S4).

Previous microarray-based studies searching for RA-responsive genes have not made the distinction between direct and indirect target genes or different germ layers. Chen and colleagues (Zhang et al., 2013a) made use of whole embryos with downregulated RA signalling. In the same study, *Hnf1b* was listed as one of 138 genes downregulated by at least 2-fold in either stage 12, 23 or 34, although no further analyses were reported. The global screen for RAR-responsive genes performed by Blumberg and colleagues (Arima et al., 2005) used stage 18 embryos. There is an obvious overlap between the genes identified in these two previous screens and the current results, for example in respect to the Hox gene family.

RA target genes involved in pancreas specification are expected to be expressed in the dorsal endoderm of gastrula-stage embryos (Chalmers and Slack, 2000; Chen et al., 2004). WMISH analysis of the expression characteristics of the 22 primary candidate genes defined above revealed two genes with these expression characteristics, namely *Hnf1b* and *Fzd4* (Fig. 2C,D). The other RA target genes were found to be expressed in the outer and/or internal involuting mesoderm, with some also expressed in the prospective neuroectoderm (Fig. S3).

*Hnf1b* was detected throughout the endoderm, with a slight enrichment in the dorsal area, as also confirmed by Nanostring analysis (Fig. 2D, Fig. S4). *Fzd4* was most predominantly expressed in the territory of the endodermal pancreatic precursor cell population, as well as in the prospective neuroectoderm (Fig. 2D). Upon decreased endogenous RA signalling by *Cyp26a1* RNA injection or by treatment with the RA-signalling inhibitor BMS453, both genes showed strongly reduced endodermal expression, upon treatment with exogenous RA increased expression (Fig. S5), confirming the *in vivo* relevance of RA signalling for the transcriptional control of both genes.

In summary, the screen for RA-induced genes with possible relevance for early events in pancreas development identified genes encoding the putative Wnt-receptor Fzd4 and the homeobox transcription factor Hnf1β. Both genes were expressed in the territory of gastrula-stage embryos where pancreatic precursor cells form and both were positively regulated by RA both *in vitro* and *in vivo*.

### Hnf1β is required for pancreas development *in vitro* and *in vivo*

**Fig. 3.**
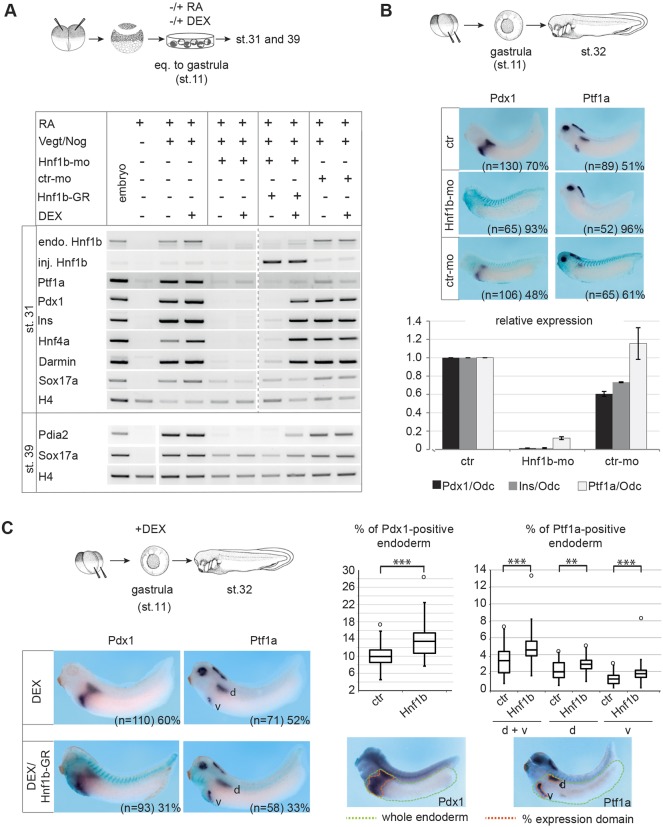
***Hnf1b* is required for pancreas development *in vitro* and *in vivo*.** (A) MO-mediated knockdown of *Hnf1b* in pancreatic explants. To demonstrate the specificity of the MO effect, RNA encoding a hormone-inducible version of Hnf1β (Hnf1b-GR) was co-injected and explants were treated with the GR inducer dexamethasone (DEX) together with RA at the equivalent of the gastrula stage. At the equivalent of stages 31 and 39, total RNA was isolated from ∼30 explants per condition and subjected to RT-PCR. Detection was of endogenous (endo) and injected *Hnf1b* (inj.), as well as for the marker genes indicated. The *Hnf1b* loss-of-function phenotype and its rescue was observed for four independent biological replicates. (B) Four-cell stage embryos were injected with RNA encoding β-galactosidase (glb1) and either *Hnf1b*-MO or a control-MO. At stage 32, embryos from two independent biological replicates were used for WMISH against *Pdx1* and *Ptf1a* and a real-time PCR analysis for *Pdx1*, *Ptf1a* and *Insulin*. The graph indicates the fold change in tested markers in relation to Odc. ctr, uninjected embryos. (C) Four-cell stage embryos were injected with RNA encoding β-galactosidase alone or in combination with *Hnf1b*-GR RNA. At the gastrula stage, embryos were treated with dexamethasone (DEX) to induce Hnf1β function. WMISH against *Pdx1* and *Ptf1a* at stage 32 is shown. Boxplots display the range of the percentage area of Pdx1 and endodermal Ptf1a domains in the endoderm observed in embryos from two independent biological replicates (see Fig. S7). By the use of ImageJ (https://imagej.net), *Pdx1* and *Ptf1a*-positive areas were measured (orange dotted line) as a ratio of the whole endoderm (green dotted line). Values above the upper whisker, which is set at 1.5× interquartile range above the third quartile, are indicated as maximum outliers (°). (*P*-values in an unpaired Student's *t*-test **<0.01, ***<0.001).

To examine whether Hnf1β mediates RA signalling in pancreas specification, we used an antisense Morpholino oligonucleotide (MO) affecting the splicing of *Hnf1b* heterogenous nuclear RNA (hnRNA), resulting in the production of a shortened protein that lacked both the DNA-binding and transactivation domains (Fig. S6). The downregulation of *Hnf1b* in the explant system led to a strong decrease in the expression of the pancreatic progenitor marker genes *Ptf1a* and *Pdx1*, as detected by RT-PCR analysis (Fig. 3A). Transcripts of the endo- and exocrine differentiation marker genes *Insulin* and *Pdia2* were also strongly downregulated. Surprisingly, the expression of the endodermal marker gene *Darmin* was also significantly decreased, whereas that of another endodermal marker, *Sox17a*, was only slightly decreased.

To test for the specificity of these effects, we performed a rescue experiment by co-injecting RNA encoding a dexamethasone-inducible variant of HNF1β, namely Hnf1β-GR. Upon dexamethasone treatment at the equivalent of the gastrula stage, expression of *Pdx1*, *Insulin*, *Pdia2*, *Darmin*, and the known direct *Hnf1b* target gene *Hnf4a* (Thomas et al., 2001) was fully restored in MO-injected embryos (Fig. 3A). The rescue of *Ptf1a* expression resulted in weaker and more variable effects in independent experiments. The requirement of Hnf1β for the pan-endodermal marker Darmin correlated with the pan-endodermal expression of Hnf1β.

In whole embryos, downregulation of *Hnf1b* revealed correlating effects. Upon MO injection, endodermal expression of *Pdx1* and *Ptf1a* was almost completely ablated, as revealed by WMISH analysis (Fig. 3B). Quantitative real-time PCR analysis confirmed the strongly reduced *Ptf1a* and *Pdx1* expression and revealed a similar decrease in *Insulin* expression. Hence, endodermal expression of *Hnf1b* is required for pancreas specification. We also assayed for effects of *Hnf1b* loss on other endodermal organ primordia. Maintenance of Hnf1β function was critical not only for pancreas development, but also for liver and lung development (Fig. S11A). Furthermore, we examined the effect of *Hnf1b* overexpression on *Ptf1a* and *Pdx1*. WMISH analysis revealed a marked expansion of the endodermal *Pdx1* and *Ptf1a* expression domains (Fig. 3C, Fig. S7). In terms of the effects of Hnf1b overexpression on the development of other endodermal organs, a liver marker was expanded and a lung marker was reduced, whereas *Darmin* expression was not affected (Fig. S11B). Given that both loss and gain of Hnf1β function influence the pancreatic progenitor field, we also asked whether Hnf1β can substitute for RA in pancreas specification. Thus, Hnf1β function was induced in programmed explants with blocked endogenous RA signalling. Under these conditions, *Hnf1b* rescued *Darmin* and *Hnf4a* expression, but none of the pancreatic marker genes were detected (Fig. S8).

Taken together, functional analysis of *Hnf1b* revealed that it is essential for specification of endodermal organs, including the pancreas, but that it is not the only gene that mediates the RA response in this context.

### Fzd4 is required for pancreas development *in vitro* and *in vivo*

Fzd4 occurs as two alternative splice variants: Fzd4 and Fzd4s (Yam et al., 2005; Swain et al., 2005). Fzd4 is a putative transmembrane receptor in the Wnt pathway, whereas Fzd4s lacks a transmembrane domain and, therefore, is considered a secreted protein that contains a Wnt-binding domain. Results from our RNA-seq and RT-PCR analyses suggested that both variants are directly induced by RA (Fig. S9). However, reads specific for Fzd4s were found at 20-fold lower levels compared with nondiscriminatory Fzd4/Fzd4s reads (Table S5). Nanostring and WMISH probes against Fzd4 transcripts should detect both variants; however, WMISH probes specific for Fzd4s stained the entire gastrula embryo, probably reflecting unspecific binding events (data not shown).

**Fig. 4.**
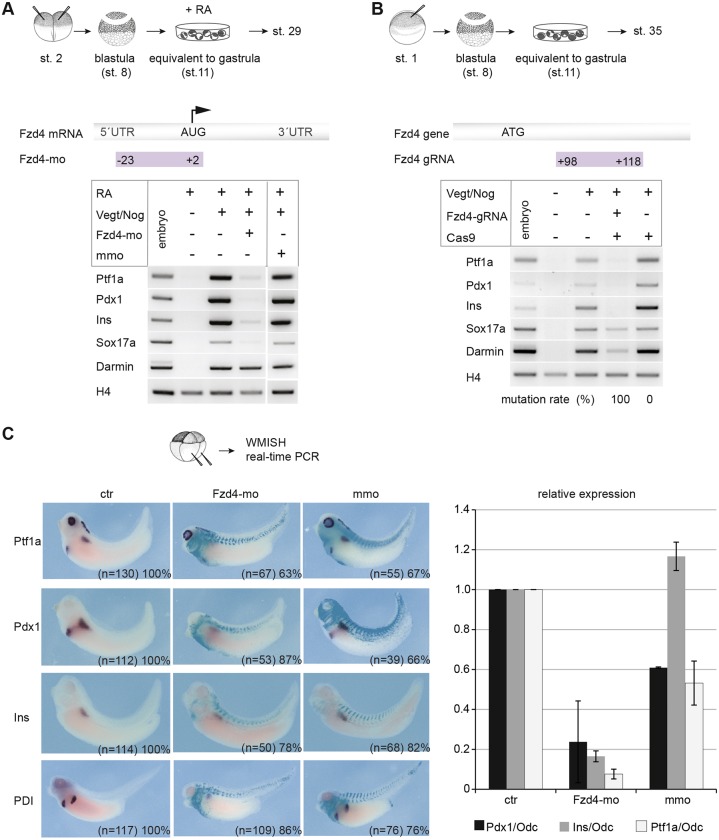
***Fzd4*/*Fzd4s* is required for pancreas development *in vitro* and *in vivo*.** (A) *Fzd4*-mo or the corresponding mismatch-MO (mmo) were co-injected along with *Vegt*- and *Noggin*-encoding RNAs. At the equivalent of stage 28, total RNA was isolated from the programmed explants and subjected to RT-PCR as indicated. (B) *Fzd4*-gRNA was co-injected along with RNAs encoding *Cas9*, *Vegt*, and *Noggin* into one-cell stage embryos. Explants were cultivated until the equivalent of stage 35. RT-PCR was for the genes indicated. Mutation rate is given for Cas9 only or for Cas9 in combination with *Fzd4*-gRNA. For both loss-of-function approaches, ∼30 explants per condition from two independent biological replicates were used. (C) Downregulation of *Fzd4*/*Fzd4s* by *Fzd4*-morpholino injection. 4-cell stage embryos were injected with RNA coding for β-galactosidase (glb1) and either *Fzd4*/*Fzd4s*-mo or the corresponding mmo. At stage 35/39, embryos from two independent biological replicates were used for WMISH against the indicated pancreatic markers and a real-time PCR analysis for *Pdx1*, *Ptf1a* and *Insulin*. The graph indicates the fold change in tested markers in relation to *Odc*. Average values are given as mean and error bars as s.e.m. ctr, uninjected embryos.

To examine whether Fzd4/Fzd4s have a regulatory function in pancreas development, we downregulated the expression of both variants by using an MO antisense oligonucleotide that blocks translation (as described by Gorny et al., 2013). Upon *Fzd4*/*Fzd4s* knockdown in pancreatic explants, *Ptf1a*, *Pdx1*, *Insulin* and *Sox17a* expression was lost compared with mismatch control MO-injected samples (Fig. 4A). Reduced pancreatic progenitor as well as differentiation marker gene expression was also observed in embryos and confirmed by real-time PCR (Fig. 4C). Furthermore, CRISPR/Cas-mediated gene mutations were used as an alternative loss-of-function approach in the explant system. Upon Fzd4-gRNA co-injection, a phenotype similar to that resulting from MO-mediated knockdown was observed (Fig. 4B). DNA sequence analysis revealed a 100% mutation rate in the *Fzd4* exon1, resulting in deletions and sequence alterations in the Fzd4/Fzd4s proteins (Fig. S10A). Potential off-targets were predicted as described (Stemmer et al., 2015) (Table S6) and mutations were not detected (Fig. S10B). We also tested the effects of downregulating Fzd4 function on the development of other endodermal organs; this resulted in the reduced expression of lung and liver markers, whereas the expression of a stomach/intestine marker expanded into more anterior territories (Fig. S12).

Taken together, both loss-of-function approaches revealed a requirement of Fzd4 and/or Fzd4s for endodermal patterning for the formation of different organs, including the pancreas, in *Xenopus*.

## DISCUSSION

**Fig. 5.**
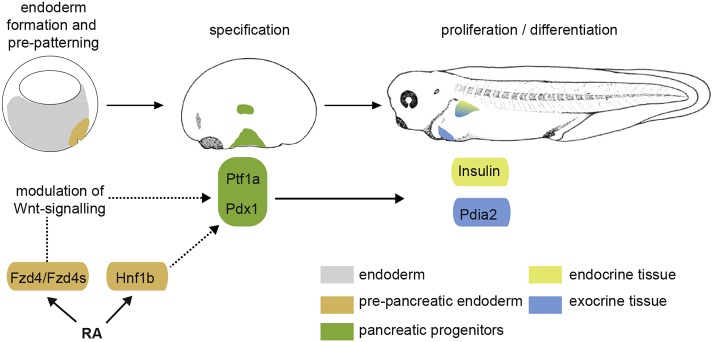
**Diagrammatic representation reflecting the role of RA signalling in pancreas specification during early *Xenopus* embryogenesis.** During gastrulation, the expression of *Fzd4* and *Hnf1b* is directly induced by RA. The overlapping activity of *Fzd4* and *Hnf1b* establishes a pre-pancreatic domain within the dorsal endoderm. *Fzd4*/*Fzd4s* is a regulator of Wnt signalling within the dorsal endoderm, which modulates Wnt signalling to a level that allows the specification of pancreatic progenitors characterised by the co-expression of *Ptf1a* and *Pdx1*. Pancreatic progenitors subsequently proliferate and differentiate into endocrine and exocrine tissue.

VegT/Noggin-programmed ectodermal explants from *Xenopus* embryos recapitulate the molecular events of pancreas specification and differentiation in a RA-dependent manner. By using this system, *Hnf1b* and *Fzd4* were identified as direct RA target genes in the context of pancreas specification. Both genes appear to be involved in pre-patterning events of the embryonic endoderm that allow for pancreas formation. Hnf1β is a homeobox transcription factor functioning upstream of Pdx1 and Ptf1a, whereas Fzd4/Fzd4s appears to serve as a Wnt modulator, establishing adequate levels of Wnt signalling to allow for pancreas development (as shown schematically in Fig. 5).

### Programming ectodermal explants to a pancreatic fate

The *Xenopus* explant system has been used to generate pancreatic tissue by the utilisation of simple protocols, including an endoderm-inducing factor and RA (Moriya et al., 2000b; Chen et al., 2004). Protocols for the generation of pancreatic tissue from hESCs or iPSCs similarly include RA, even though such multistep protocols are more complex and include multiple signalling molecules (Pagliuca et al., 2014; Shaer et al., 2015).

In the current study, we used the maternal transcription factor VegT to promote endoderm formation and created a dorsal endodermal environment by application of the BMP inhibitor Noggin. With the addition of RA, we aimed to recapitulate *in vivo* RA secretion from the dorsal mesoderm during gastrulation. The temporal profile of pancreatic progenitor and differentiation marker gene expression in such explants correlated with the pattern observed for pancreas development in whole embryos. These findings open the way for the use of pancreatic explants in studies aiming to elucidate the molecular mechanisms behind pancreas specification induced by RA.

However, there are some limitations of this explant system. As revealed by WMISH, the yield of pancreatic cells varied significantly and was never complete. A major portion of the cells either adopted a different endodermal state or developed a mesodermal or neuroectodermal fate, which could be indirectly induced by the activity of VegT. In line with such results, we observed robust expression of mesodermal and neuroectodermal marker genes, even though their level of expression was lower than that of general endodermal and pancreatic genes.

### *Hnf1b* is a direct RA target required for pancreas development

We identified *Hnf1b* as a direct RA-responsive gene expressed in the early embryonic endoderm. Induction of *Hnf1b* by RA had previously been described for the murine hindbrain (Sirbu and Duester, 2006). In the explant system used in the current study, we found *Hnf1b* to be expressed in a RA-dependent manner. The idea of RA responsiveness was further supported by the prediction of two RA-responsive elements (RAREs) in the genomic locus of the mouse *Hnf1b* gene (Power and Cereghini, 1996; Pouilhe et al., 2007).

RA-induced expression of *Hnf1b* in the dorsal endoderm of gastrula-stage embryos appears to have a role in endoderm patterning. Our functional analyses in programmed explants and whole embryos revealed an essential role for *Hnf1b* in pancreas development, correlating with a loss of *Ptf1a* and *Pdx1* expression. These findings are consistent with observations in *HNF1b*-mutant mice (Haumaitre et al., 2005). Interestingly, Maestro et al. (2003) defined *Hnf1b*-positive cells as a cellular stage distinct from *Pdx1*/*p48* (*Acot10* – Mouse Genome Informatics) multipotent pre-pancreatic cells in the mouse. Although we agree with defining this endodermal field as containing multipotent pancreatic cells in *Xenopus*, on the basis of the overlap of *Hnf1b* with *Pdx1* expression domains in gastrula-stage endoderm, Hnf1β-positive cells do not yet express *p48* (*Ptf1a*). It also appears that, in *Xenopus*, the onset of expression of *Pdx1* and *Hnf1b* occurs simultaneously, in principle. Humans with a monogenetic form of diabetes, referred to as maturity onset diabetes of the young 5 (MODY 5) (Horikawa et al., 1997), are mutant in *HNF1B*. A previous study identified Hnf1β as a downstream target for RA in the context of zebrafish pancreas development (Song et al., 2007). However, direct induction of Hnf1β by RA and the early requirement of Hnf1β during gastrula and/or neurula stages were not revealed in this previous study.

In gastrula-stage embryos, RA-responsive *Hnf1b* expression was restricted to the dorsal endoderm. *Hnf1b* also exhibits RA-independent, pan-endodermal expression during early embryogenesis (Demartis et al., 1994); Hnf1β was previously identified as a direct downstream target of Sox17α, a regulator of endoderm development, which is expressed in an overlapping manner (Hudson et al., 1997; Clements et al., 1999; Sinner et al., 2004). Pan-endodermal expression has also been described for Darmin, a putative metalloprotease (Pera et al., 2003). Similar to Hnf1β, expression of *Darmin* was RA responsive in the explant system. We further observed a loss of *Darmin* expression upon Hnf1β downregulation, as well as Hnf1β-triggered induction of *Darmin* in RA-ablated explants. These observations clearly revealed *Darmin* to be a gene that occurs downstream of *Hnf1b*.

*Hnf1b* overexpression resulted in a modest expansion of the pancreatic progenitor domain, but this was not sufficient for ectopic pancreatic progenitor gene expression elsewhere in the endoderm, comparable to what has been previously described following combined ectopic expression of *Ptf1a* and *Pdx1* (Afelik et al., 2006). Furthermore, Hnf1β alone was not sufficient to substitute RA in the induction of pancreatic marker genes in the explant system, suggesting the requirement for one or more additional RA-regulated factors to allow for induction of a pancreatic fate. We also detected effects of modulating Hnf1β activity on the development of other endodermal organs, perhaps reflecting a more general role for Hnf1β in this context. Such a function correlates with the pan-endodermal expression of *Hnf1b* in gastrula-stage embryos.

### *Fzd4*/*Fzd4s* is a direct RA target gene required for pancreas development

Expression of Fzd4 during gastrulation was initially described for the prospective neuroectoderm (Shi and Boucaut, 2000). We observed endodermal expression of *Fzd4* in RA-treated embryos and also in untreated embryos upon prolonged staining. In the pancreatic explant system, we identified *Fzd4* as a direct RA target gene. Co-expression of two alternative splice variants, the transmembrane form Fzd4 and the secreted soluble form Fzd4s, has been described for *Xenopus* embryos (Sagara et al., 2001; Yam et al., 2005; Swain et al., 2005). In our RNA-seq experiments, a significant number of mapped reads for *Fzd4*/*Fzd4s* were induced by RA in the presence of a translational inhibitor, indicating that the expression of both *Fzd4* variants is directly regulated by RA. Fzd4s-specific reads appeared at a lower level compared with the number of nondiscriminatory *Fzd4*/*Fzd4s* reads, indicating that the transmembrane version of *Fzd4* is predominantly expressed. It has been reported that *Fzd4* mRNA is provided maternally with increasing expression during gastrulation, whereas *Fzd4s* expression is only initiated during gastrulation (Swain et al., 2005). Remarkably, *Fzd4*/*Fzd4s* expression overlapped with the territory of putative pancreatic precursor cells in the dorsal endoderm, adjacent to the expression domain of the RA-generating enzyme RALDH2 in the dorsal mesoderm (Chen et al., 2001).

Previous loss-of-function studies with *Fzd4*/*Fzd4s* in *Xenopus* revealed defects in fin formation and neural crest migration (Gorny et al., 2013). Comparative transcriptome analyses in the mouse identified *Fzd4* transcripts to be enriched in pancreatic progenitor cells compared with liver progenitors, although a function of *Fzd4*/*Fzd4s* in pancreas development was not demonstrated experimentally (Rodriguez-Seguel et al., 2013). Using two independent loss-of-function approaches, we found that *Fzd4*/*Fzd4s* is required for pancreas specification and differentiation in programmed *Xenopus* explants. Results obtained upon downregulation of *Fzd4*/*Fzd4s* in whole embryos provided further support for this notion, because significantly reduced levels of pancreatic progenitor and differentiation marker gene expression were observed.

### *Fzd4*/*Fzd4s* as modulators of Wnt signalling in pancreas development

It was proposed by several previous studies that both canonical and noncanonical Wnt-signalling activities must be precisely controlled in the anterior endoderm of early embryos to maintain foregut identity and, thus, allow endodermal patterning appropriate for endodermal organ formation (McLin et al., 2007; Li et al., 2008; Damianitsch et al., 2009; Zhang et al., 2013b; Rodriguez-Seguel et al., 2013). *Fzd4* appears to define an activity that is also involved in this process. It encodes a transmembrane protein described to function as a receptor in both canonical and noncanonical Wnt signalling downstream of Wnt5a (Umbhauer et al., 2000; Mikels and Nusse, 2006). Conversely, Fzd4s exhibits structural similarities with secreted frizzled related proteins (Rattner et al., 1997) and was demonstrated to bind Wnt ligands, thereby functioning as an activator and/or inhibitor of canonical Wnt signalling, depending on the identity of a given Wnt ligand (Sagara et al., 2001; Swain et al., 2005; Gorny et al., 2013). Another endodermally expressed Wnt receptor, Fzd7, was shown to modulate canonical as well as noncanonical Wnt signalling positively or negatively in a concentration-dependent manner, promoting foregut fate and thereby pancreas development. The loss of Fzd7 was shown to result in agenesis of liver and pancreas, with an overall loss of foregut identity (Zhang et al., 2013b). We observed a similar foregut phenotype upon *Fzd4*/*Fzd4s* downregulation *in vivo*. Thus, a precisely regulated concentration of the transmembrane receptor Fzd4 and/or secreted Fzd4s might be required to allow for pancreas development in the foregut. To obtain such concentration levels at the appropriate time during endoderm development, a time-controlled expression system for Fzd4/Fzd4s would be required. However, our initial attempts using a Tet-inducible system have not yet been successful. In summary, we propose that Fzd4/Fzd4s functions as a modulator of Wnt signalling downstream of RA in the context of endodermal patterning that allows for pancreas development in *Xenopus*.

## MATERIALS AND METHODS

### Microinjections and ectodermal explants

*Xenopus laevis* embryos were obtained by *in vitro* fertilisation and staged according to Nieuwkoop and Faber (1967). Capped sense RNAs were transcribed using the mMessage mMachine kits (Ambion). The following linearisation and transcription conditions were used with indicated amounts of RNA injected per embryo: VegtT (*Not*I/Sp6; 500 pg; Zhang and King, 1996), Noggin (*Not*I/Sp6; 500 pg; Smith et al., 1993), Cyp26a1 (*Mlu*I/T3; 2 ng; Hollemann et al., 1998), Hnf1β-GR (*Not*I/Sp6; 800 pg; Vignali et al., 2000), GFP (*Not*I/Sp6; 200 pg; Rubenstein et al., 1997) and β-galactosidase (*Not*I/Sp6; 200 pg; Chitnis et al., 1995). RNAs were injected in a volume of 4 nl as follows: one-cell stage animally (Fig. 4B; Fig. S10), two of two blastomeres animally (Fig. 1; Fig. 2; Fig. 3A; Fig. 4A; Fig. S1; Fig. S2A; Fig. S6; Fig. S8; Fig. S9), two of four blastomeres vegetally dorsal (Fig. 3B/C; Fig. S7; Fig. S11) or animally and vegetally dorsal (Fig. S2B; Fig. S4) or marginally dorsal (Fig. S5A), and two of eight vegetally dorsal (Fig. 4C; Fig. S12). Ectodermal explants were dissected from the blastocoel roof of stage 8/9 embryos and cultured in salt solution [88 mM NaCl, 1 mM KCl, 0.82 mM MgSO_4_, 2.4 mM NaHCO_3_, 0.41 mM CaCl_2_, 0.33 mM Ca(NO_3_)_2_, 10 mM HEPES, pH 7.8] with antibiotics [ampicillin (100 µg/ml), kanamycin (10 µg/ml) and gentamycin (10 µg/ml), ROTH] on 0.7% agarose at 14°C until control embryos had reached the desired stage.

### Morpholino oligonucleotides and CRISPR/Cas

For knockdown experiments, antisense HNF1β-MO (CCTCGCTGTGAACAAAA CACAAA; 25 ng/embryo for WMISH/qPCR and 55 ng/embryo for explants), control-MO (CCTCTTACCTCAGTTACAATTTATA; same amounts as for HNF1β-MO), Fzd4/Fzd4s-MO (Gorny et al., 2013; ATTATTCTTCTTCTGTTGCCG CTGA; 5 ng/embryo for WMISH/qPCR and 45 ng/embryo for explants) and Fzd4-mismatch-MO (ATTATTaTTaTTCTaTTGCaGCTaA; same amounts as for Fzd4/Fzd4s-MO). For knockout experiments using CRISPR/Cas, 3 ng/embryo capped sense RNA of Cas9 (Blitz et al., 2013), prepared by Acc651 linearisation and transcribed with the mMessage mMachine T7 kit (Ambion), was injected animally at the one-cell stage alone or together with 300 pg/embryo uncapped sense Fzd4-gRNA, linearised with *Dra*I and transcribed with a MEGAscript T7 kit (Invitrogen). Fzd4-gRNA was generated by cloning the oligonucleotides 5′phosp-TAGGCACATGGTGATCCTGATG and 5′phosp-AAACCATCAGGATCACCATGTG into pDR274 (Addgene). For target- and predicted potential off-target (CRISPR/Cas Target online predictor; CCTop; Stemmer et al., 2015) mutation analysis, genomic DNA from 50 explants, per condition and biological replicate, was isolated by using a ‘DNeasy Blood and Tissue Kit’ (Qiagen). The region around the target site and predicted off-target sites was amplified and cloned into the pGem^®^-T Easy vector (Promega) for sequence analysis.

### Chemical treatments of embryos and explants

Treatments with 5 µM RA (all-*trans* RA, SIGMA) (variations are indicated) were done in the corresponding buffer at embryonic stage 11 for 1 h at 12°C under light protection. After the treatment, embryos and explants were cultivated until the controls reached the desired developmental stage. Treatments with 10 µg/ml Cyclohexamide (SIGMA) started 30 min prior to additional treatments. Whole embryos were incubated with 0.25 µM BMS453 (a gift from Bristol Myers Squibb; Chen et al., 1995) at stage 8/9 until stage 12. Afterwards, embryos were cultivated until they reached the desired stage. Injected embryos or ectodermal explants were cultivated in the dark in the presence of 10 µM dexamethasone (Sigma) at the equivalent of stage 11 until the desired stage was reached.

### Whole-mount *in situ* hybridisation

WMISH of whole embryos and explants was done as previously described (Harland, 1991) with modifications (Hollemann and Pieler, 1999). The probes were prepared as follows: Cebpd (*EcoR*I/T7; Ikuzawa et al., 2005), Cyp26a1 (*Cla*I/T7; Hollemann et al., 1998), Darmin (*EcoR*I/T7; Pera et al., 2003), Dhrs3 (*EcoR*I/SP6; Kam et al., 2010), Foxh1 (*EcoR*I/T7; Kofron et al., 2004), IFABP (*Nco*I/Sp6; Henry et al., 1996), Fst (*Sal*I/T7; Tashiro et al., 1991), Fzd4 (*BamH*I/T7; Swain et al., 2005), Gbx2 (*Apa*I/SP6; Maczkowiak et al., 2010), hHex (*Not*I/T7; Newman et al., 1997), Hnf1β (*BamH*I/T7; Vignali et al., 2000), Hoxa1-b (*Sal*I/T7; Sive and Cheng, 1991), Hoxb1 (*EcoR*I/SP6; Nieto et al., 1992), Hoxd1 (*EcoR*I/T7; Sive and Cheng, 1991), Hoxd4 (*EcoR*I/T7; Klein et al., 2002), Igf3 (*Sal*I/T7; Richard-Parpaillon et al., 2002), Ins (*Not*I/T7; Shuldiner et al., 1989), Lhx1 (*Xho*I/T7; Taira et al., 1994), Meis3a (*Cla*I/T3; Salzberg et al., 1999), Nkx2.1 (*No*tI/T7; Hollemann and Pieler, 2000), Nkx6.2 (*Xho*I/T7; Dichmann and Harland, 2011), Pdia2 (*BamH*I/T7; Sogame et al., 2003), Pdx1 (*Apa*I/SP6; Wright et al., 1989), Prph (*Sal*I/T7; Sharpe et al., 1989), Ptf1a (*Not*I/T7; Afelik et al., 2006), Xl.45046 (*Sal*I/T7; BioScience IMAGp998J07121170Q), Xl.47239 (*Sma*I/T7; BioScience IRBHp990G0486), Xl.51509 (*Sal*I/T7; BioScience IMAGp998L11929 6Q) and Xl.57926 (*Cla*I/T7; BioScience IMAGp998C1718900Q).

### RT-PCR and real-time RT-PCR

Total RNA from whole embryos and explants was isolated using the peqGOLD Trifast reagent (peQlab) and reverse transcribed by the use of random hexamer oligonucleotides (Invitrogen) and MuLV reverse transcriptase (Roche). The following oligonucleotides were used for target amplification in RT-PCR: injected CYP26a1 (GTCGACCTGTGGATCCAAAGA/GATGCGTCTTGTAGATGCGAC); endogenous CYP26a1 (CCCGGAGATTCCTCGAGGTT/GACACCACGACCAAGACCCG); Darmin (GGTTACCGATTACTTGGAGG/AGCATCATCTGGTCCACCAA); Fzd4s (CATCAGGATCACCATGTGCCAG/GAAAGTAAACCCCCTGTGCTGAG); Glucagon (AGAATTTATTGAGTGGTTGA/ATCGGCATGTCTTCTGTCC); H4 (CGGGATAACATTCAGGGTATCACT/ATCCATGGCGGTAACTGTCTTCCT); injected HNF1β (CG GGGACATGTGCAAGTTCT/CAAGCTACTTGTTCTTTTTGC); HNF1β (AAAGGGCAGAAGT GGACAGG/ATGCAGCACGTTTTTGGGTC); Hnf4α (AGACTCCCCAACCATCTCCA/CGCTTTCCCAAAGAGGCAAC); Insulin (ATGGCTCTATGGATGCAGTG/AGAGAACATGTGCTGT GGCA); Odc (GCCATTGTGAAGACTCTCTCCATTC/TTCGGGTGATTCCTTGCCAC); Pdx1 (GTCCTCCAGACATCTCACCG/AGCATGACTGCCAGCTCTAC); Pdia2 (GGAGGAAAGAGG GACCAA/GCGCCAGGGCAAAAGTG); Ptf1a (GTTGTCAGAACGGCCAAAGT/GGTACCGAGTGGAACCAAAG); and Sox17α (CAAGAGACTGGCACAGCAGA/CTGCTTGGGGTTCCCTGTAG). Oligonucleotides that were different for real-time PCR were as follows: Pdx1 (GTCCTCCAGACATCTCA CCG/AGCATGACTGCCAGCTCTAC) and Ptf1a (GGTACAGTCCGATCTGCCGC/GGAGTCCAC ACTTTGGCCGT). The iQ SYBR Green Supermix (Bio-Rad) and the iCycler iQ detection system (Bio-Rad) were used for measurements. Relative expression was calculated by normalising to the expression levels of Odc. Both RT- and real-time RT-PCR were done for at least two independent biological replicates. Error bars result from the standard error of the mean (s.e.m.).

### Library preparation for RNA sequencing

Library preparation for RNA sequencing was performed using the TruSeq Stranded Total RNA with Ribo-Zero Gold kit removing both cytoplasmic and mitochondrial rRNA (Illumina, Cat. No. RS-122-2201). RNA samples from two independent experiments with ∼50 explants for each condition and ∼200 ng of total RNA were used as starting material. Accurate quantitation of cDNA libraries was performed by using the QuantiFluor™ dsDNA System (Promega). The size range of final cDNA libraries was determined by applying the DNA 1000 chip on the Bioanalyzer 2100 from Agilent (280 bp). cDNA libraries were amplified and sequenced by using the cBot and HiSeq2000 from Illumina with 50 bp single-end chemistry.

### Data pre-processing and bioinformatics analysis

The sequence intensity images were transformed to bcl files (BaseCaller) and were demultiplexed to fastq files with CASAVA (version 1.8.2). The quality of resulting sequence reads was checked by FastQC (http://www.bioinformatics.babraham.ac.uk/projects/fastqc/). Obtained sequence reads were aligned to the transcript reference sequences of *Xenopus tropicalis* (kindly provided by Michael J. Gilchrist; Gilchrist et al., 2004). Reads that could not be characterised were also aligned to selected *X. laevis* transcriptome sequences (UniGene). Alignment was performed using Bowtie2 (version 2.1.0) in local alignment mode allowing six mismatches within 50 bases (Langmead and Salzberg, 2012). Conversion of resulting alignment files, sorting, filtering of unique hits and counting were conducted with samtools (Li et al., 2009). Read count data were analysed in the R/Bioconductor environment (http://www.bioconductor.org) loading edgeR (Robinson et al., 2010). Counts were normalised to trimmed mean of M-values and the dispersion was estimated. For the detection of differentially expressed genes, a test based on a generalised linear model likelihood ratio assuming negative binominal data distribution was performed via edgeR. Candidate genes were filtered to a minimum of a 2-fold change difference from the control and a FDR-corrected *P*-value of <0.05. For transcript-specific determination of Fzd4/Fzd4s abundances, the reads were aligned to the *X. laevis* genome version Xenla9.1 using the STAR alignment software (Dobin et al., 2013) allowing for two mismatches. Reads mapping to the whole *Fzd4* gene region (Fzd4/Fzd4s) and the annotated *Fzd4* intron only (Fzd4s) were counted using the bedtools coverage command (http://bedtools.readthedocs.io).

### Nanostring analysis

For the Nanostring analysis, 600 ng of total RNA from five embryos or 50 explants was used. The counts were normalised in two steps using the nSolver software (https://www.nanostring.com/products/analysis-software/nsolver). The counts were initially normalised with respect to the mean of positive control counts and then normalised to the geometric mean of the housekeeping gene encoding ornithine carboxylase (*odc*). Finally, to consider the background, the mean and 2-fold of the standard deviation of the eight negative controls were subtracted. Values less than 1 were set to 1. Data from two independent experiments (A and B) were used to calculate a mean value. Error bars indicate the s.e.m.

## Supplementary Material

Supplementary information
