# Supplementary material for: Retinoic acid-induced expression of Hnf1b and Fzd4 is required for pancreas development in Xenopus laevis
Source: Development. 2018 Jun 8;145(12):dev161372. doi: 10.1242/dev.161372 (PMC6031401; doi:10.1242/dev.161372)
Supplement: Supplementary information [file develop-145-161372-s1.pdf]

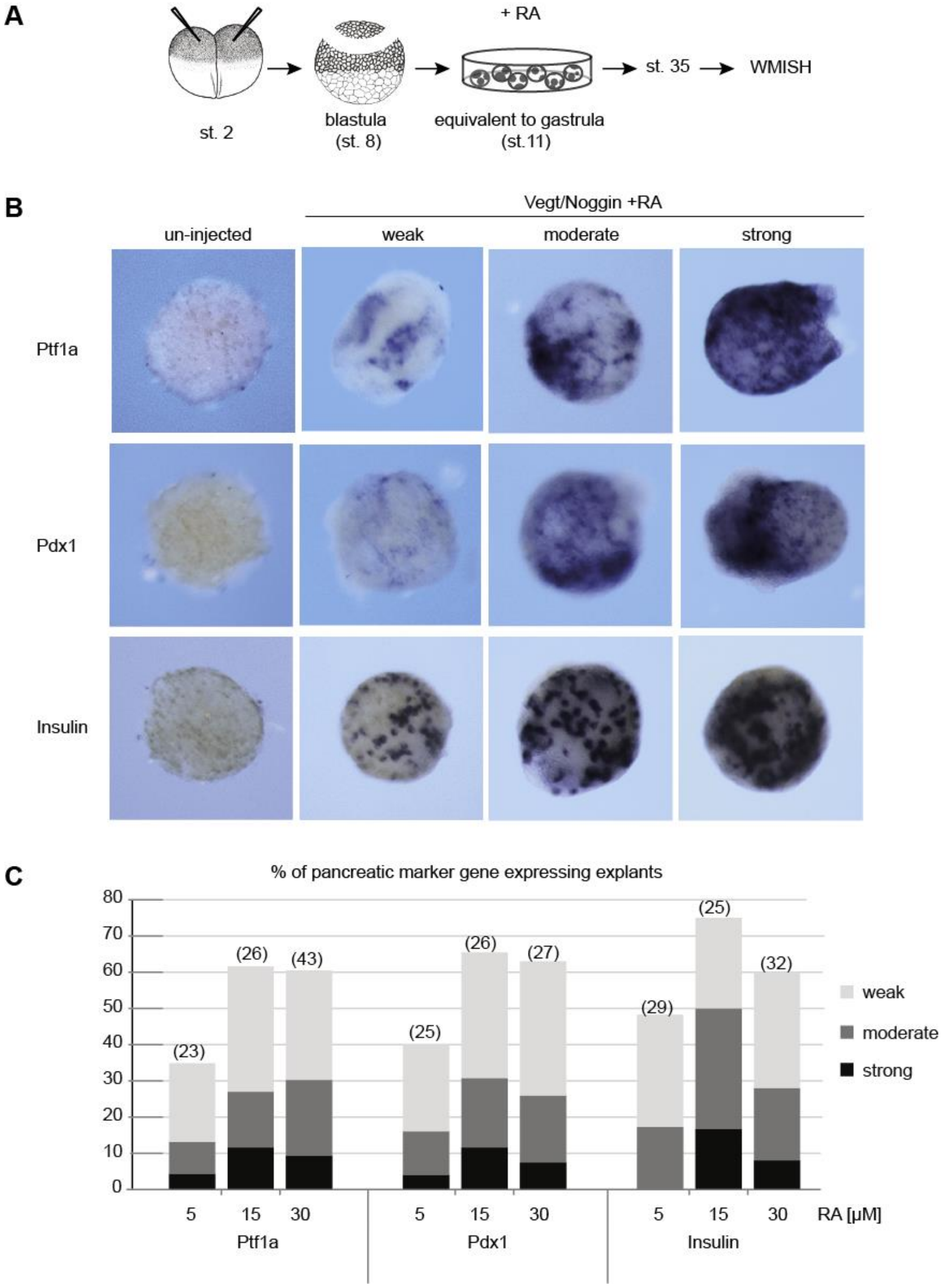

Fig. S1. Distribution of pancreatic cells in programmed explants

(A) Co-injection of *vegt* and *noggin* mRNAs into the animal pole of two cell stage embryos. Explants were treated with three different concentrations of RA (5, 15 and 30µM) at the equivalent of stage 11 for 1h and used for WMISH at the equivalent of stage 35. (B) Different categories according to the intensity of pancreatic marker gene expression are presented. (C) Diagram reflecting the percentage of explants positive for pancreatic marker gene expression in the different categories. The total number of explants analyzed is indicated in brackets.

Development • Supplementary information

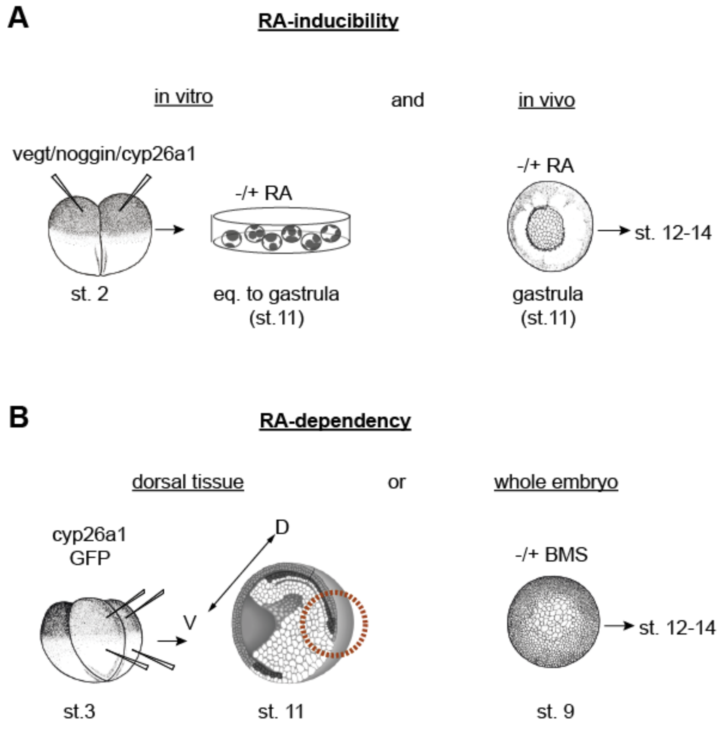

Fig. S2. Verification of RA-responsiveness

(A) Ectodermal explants from *vegt/nog/cyp26a1*-injected embryos (*in vitro*) or whole embryos (*in vivo*) were treated with RA or left untreated. Analysis of candidate gene expression was by use of the Nanostring technology. (B) RA-signalling was either impaired by Cyp26a1 RNA injection into the two dorsal blastomers at the four-cell stage and dorsal tissue dissected at stage 11 (red dotted line), or whole embryos were treated with BMS453 from stage 9 to 12/14 and analyzed using Nanostring. (C) Venn diagram illustrating the number of genes verified for their RA-inducibility *in vitro* and *in vivo* as well as for their RA-dependence *in vivo*.

Development • Supplementary information

direct	gene	expression
•	*hnf1b*	E
•	*fzd4*	NEc
		E
•	*cebpd*	IIM
•	*dhrs3*	IIM
	lhx1	IIM
	fst	IIM
•	*cyp26a1*	NEc
		OIM
•	*hoxd4*	NEc
		OIM
•	*gbx2.1*	NEc
		OIM
•	*hoxa1-b*	NEc
		OIM
•	*hoxd1*	NEc
		OIM
•	*znf703*	OIM
•	*foxh1*	OIM
•	*meis3*	OIM
•	*nkx6.2*	OIM
•	*Xl.45046*	OIM
•	*Xl.51509*	OIM
•	*hoxb1*	OIM
	prph	OIM
•	*igf3*	OIM
	Xl.47239	OIM
	Xl.57926	OIM

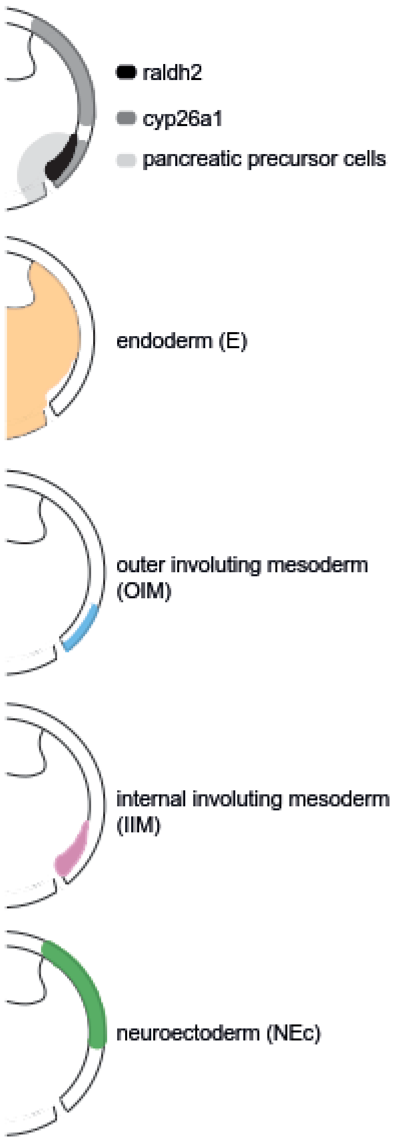

Fig. S3. Expression characteristics of RA-responsive genes at gastrula stage

Schematic overview for the expression patterns of 22 RA-responsive genes by WMISH in gastrula stage embryos. Candidate genes were grouped according to their expression domains. Dots indicate direct RA-target genes. The upmost scheme describes the expression domains of Raldh2 (RA generating enzyme), Cyp26a1 (RA-degrading enzyme) and the localization of prospective pancreatic progenitor cells. The color code reflects expression domains as indicated.

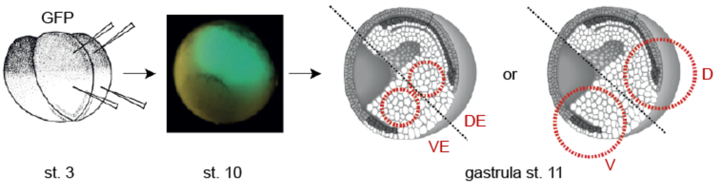

tissue	gene	CE	DE	VE	D	V
**endoderm**						
pan-	sox17a	1059	3157	3350	896	740
	darmin	497	3042	2101	129	31
dorsal	cerberus	2270	11643	2624	1543	171
**mesoderm**						
dorsal	bmp2	363	2779	812	312	156
	chordin	1628	3754	1	9973	1
ventral	bmp4	1804	1326	3224	772	2854
**ectoderm**						
epidermal	xk81a1	1293	2	9	2	139
neural	sox2	675	13	16	716	308

category	gene	CE	DE	VE	D	V
**dorsally enriched**						
	cebpd	31	291	64	36	11
	cyp26a1	1079	1264	637	1071	1583
	dhrs3	385	1034	353	676	252
	fst	90	460	131	1242	36
	fzd4	401	597	334	165	38
	hnf1b	260	965	848	212	81
	lhx1	123	392	302	449	73
	nkx6.2	30	13	1	356	7
**ventrally enriched**						
	hoxd1	1120	38	496	493	1658
	Xl.45046	172	80	246	176	298
	znf703	1485	259	838	1136	2360
**equally distributed**						
	foxh1	377	331	494	326	318
	gbx2.1	90	25	45	22	58
	hoxa1-b	56	1	2	43	93
	hoxb1	1	1	1	1	7
	hoxd4	1	1	1	2	11
	igf3	63	82	53	37	24
	meis3-a	405	304	342	330	279
	prph	21	1	1	8	5
	Xl.47239	48	34	61	30	46
	Xl.51509	16	1	1	40	60
	Xl.57926	371	240	308	580	543

Fig. S4. Nanostring analysis for the spatial expression characteristics of RA-responsive genes at gastrula stage

Four-cell stage embryos were injected into the two dorsal blastomeres with GFP mRNA. At stage 10, embryos exhibiting a GFP signal on the dorsal side were selected for further cultivation. At stage 11, either dorsal and ventral endoderm or the whole tissue surrounding the dorsal blastoporus and the corresponding ventral tissue were dissected. Nanostring counts are mean values from two independent experiments. DE = dorsal endoderm, VE = ventral endoderm, D = dorsal part, V = ventral part

Development • Supplementary information

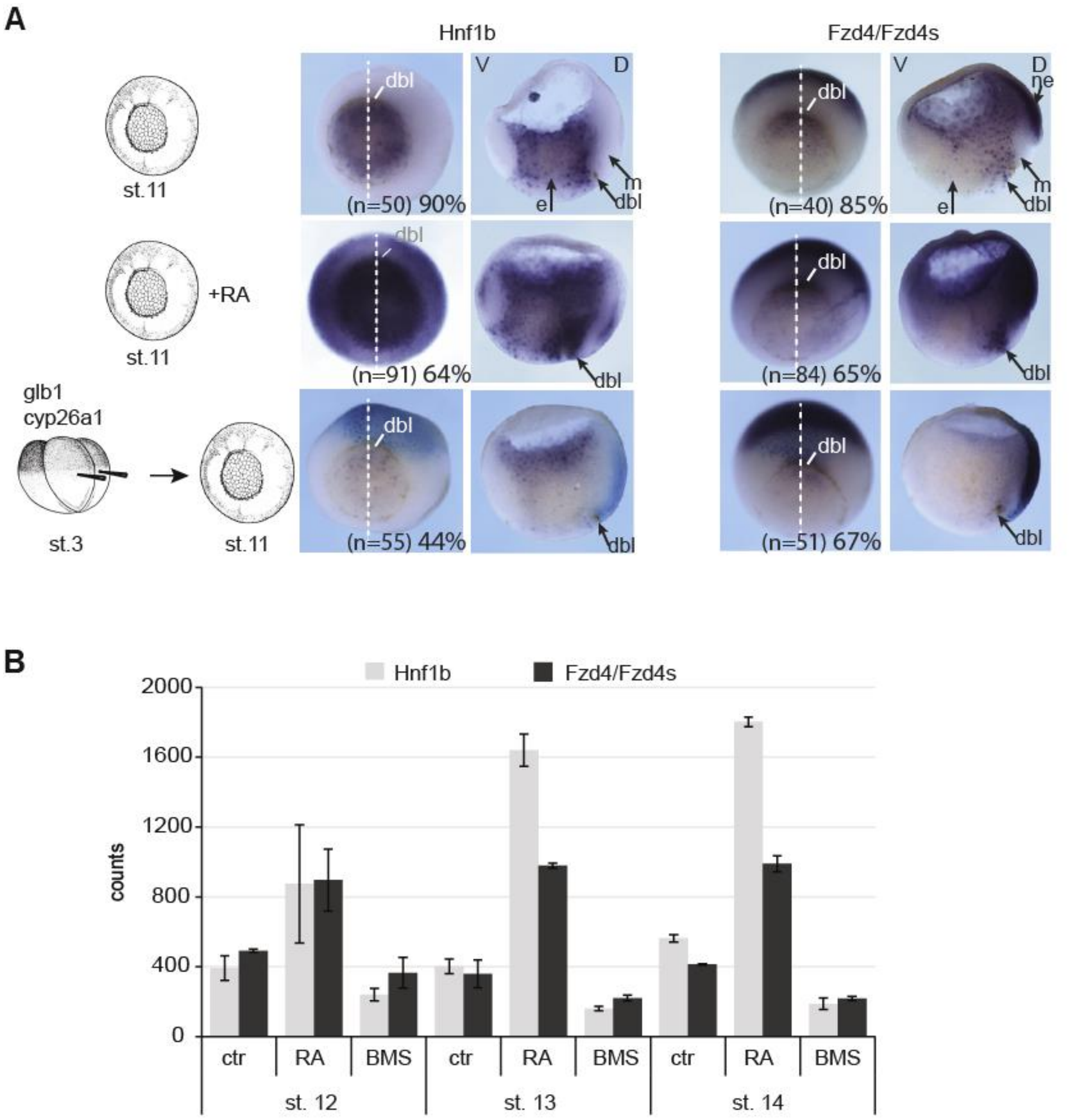

Fig. S5. Embryonic expression of Hnf1β and Fzd4 is RA-dependent

(A) WMISH for Hnf1β and Fzd4 at gastrula stage in untreated, RA-treated and *cyp26a1*-injected embryos. Images on the left display whole embryos (dorsal side up) and images on the right show bisected embryos (dorsal side on the right). dbl, dorsal blastopore lip; e, endoderm; m, mesoderm; ne, neuro-ectoderm. (B) Nanostring analysis of untreated, RA- and BMS-treated embryos collected at indicates stages.

Development • Supplementary information

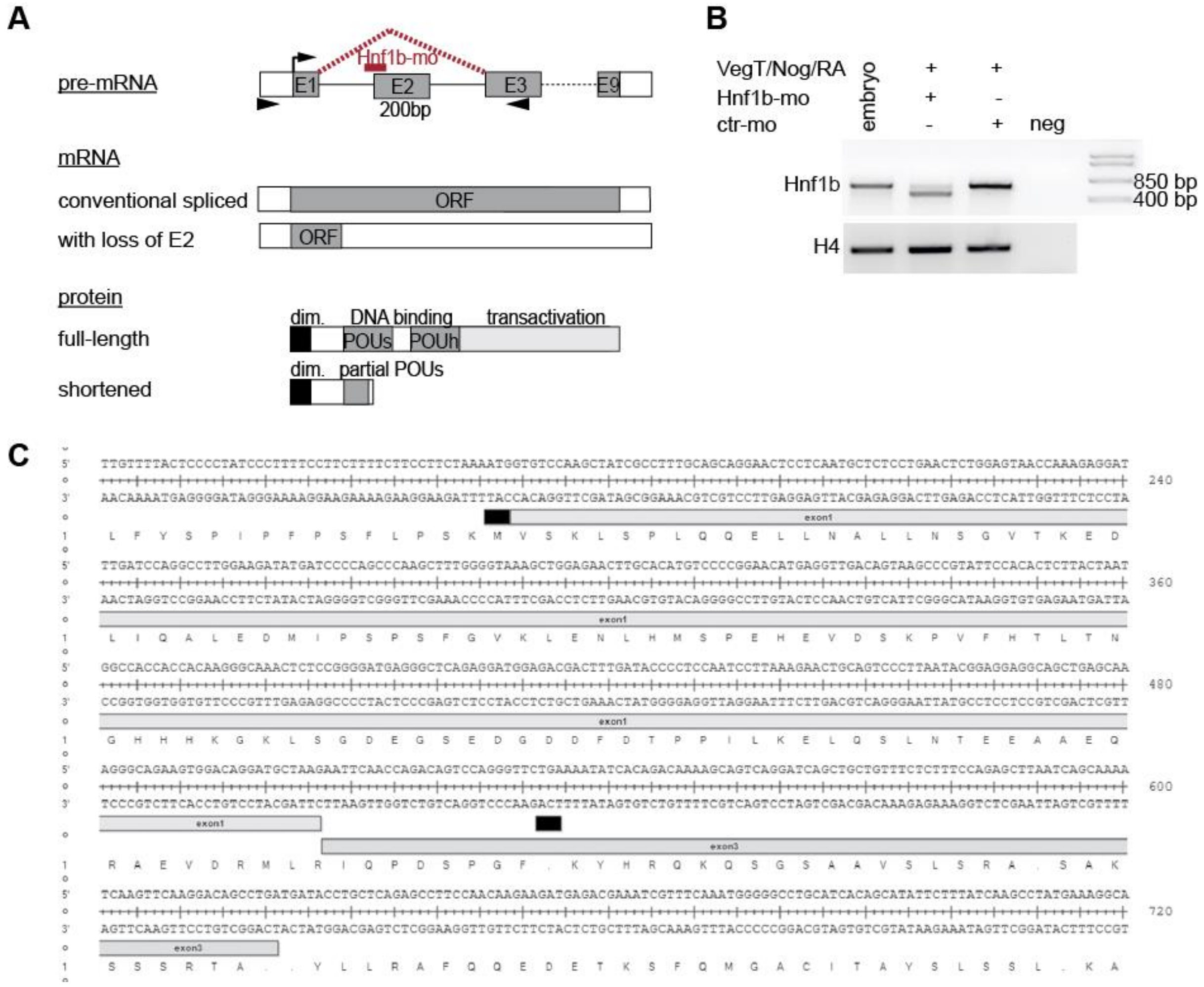

Fig. S6. Specificity of the Hnf1β morpholino antisense oligonucleotide

(A) The Hnf1β morphilono antisense oligonucleotide (Hnf1b-mo) targets the intron1/exon2 boundary (E1/E2) of the Hnf1β pre-mRNA, resulting in the loss of exon 2, leading to a shortened open reading frame (ORF) lacking functional DNA-binding domains (POU_s and POU_h) and also lacking the transactivation domain. (B) The specificity of the Morpholino was tested in the explant system. RT-PCR with oligonucleotides bind to exon 1 and exon 3. A smaller Hnf1β amplicon is detected in the presence of the morpholino. (C) Sequence analysis of the shorter Hnf1β amplicon upon Hnf1b-mo application confirms the loss of exon 2.

Development • Supplementary information

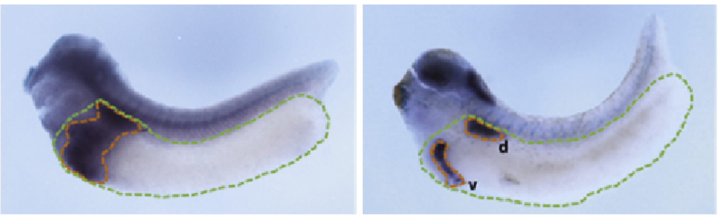

replicates	% pdx1 domain in the endoderm		replicates	% ptf1a domain in the endoderm					
	control	hnf1b		control			hnf1b		
				d	v	d+v	d	v	d+v
A	12.53	17.16	A	1.69	1.67	3.36	2.33	1.97	4.30
	16.73	21.40		1.52	1.61	3.13	2.64	1.74	4.39
	14.21	19.05		2.60	2.00	4.60	4.89	8.30	13.19
	17.20	14.84		1.43	1.27	2.69	3.50	1.82	5.31
	11.74	13.68		1.13	2.42	3.55	3.28	3.81	7.09
	10.11	20.14		0.40	1.29	1.68	1.84	3.67	5.51
	10.21	12.70		4.25	2.75	7.00	2.65	1.72	4.37
	10.68	17.27		1.33	1.95	3.29	2.71	0.00	2.71
	8.11	13.95		1.56	1.28	2.83	1.51	0.00	1.51
	12.28	9.31		2.63	1.81	4.45	0.97	2.16	3.13
	9.75	14.98		1.97	0.00	1.97	1.61	0.00	1.61
	10.38	15.40		1.00	0.00	1.00	3.46	1.62	5.07
	9.47	13.82		0.77	0.26	1.03	1.85	1.32	3.17
	11.45	8.39		1.29	0.00	1.29	2.65	1.99	4.64
	4.84	11.78		0.95	0.25	1.20	3.08	1.49	4.57
	8.83	10.69		2.93	0.67	3.60	2.68	1.70	4.37
	4.54	10.90		1.46	0.53	1.99	3.08	1.98	5.06
	5.31	12.81		0.59	0.00	0.59	2.89	1.67	4.55
	6.91	9.79		1.60	0.00	1.60	2.62	3.04	5.67
	6.58	10.71		2.01	0.29	2.30	2.33	1.52	3.85
	5.67	12.66		0.87	0.00	0.87	2.94	1.91	4.85
	7.03	14.98							
	7.67	8.71							
	7.04	7.69							
B	10.15	9.80	B	3.30	0.81	4.11	2.86	1.28	4.14
	9.55	10.07		3.19	1.65	4.84	3.22	2.71	5.93
	8.88	9.70		2.09	1.57	3.65	3.73	1.86	5.59
	11.11	14.12		2.59	0.73	3.31	3.93	1.07	5.00
	9.46	12.72		3.17	0.84	4.01	3.07	1.18	4.26
	9.80	12.34		1.38	0.58	1.96	3.01	2.82	5.83
	8.54	13.45		3.05	1.03	4.08	4.89	2.41	7.30
	8.70	9.38		2.16	0.82	2.98	3.70	3.75	7.45
	9.76	14.48		3.85	1.12	4.96	3.33	1.06	4.39
	9.93	9.73		2.56	1.28	3.83	2.41	1.66	4.07
	11.92	17.79		3.11	1.20	4.31	2.62	1.13	3.75
	10.07	16.01		3.72	1.37	5.09	1.54	1.00	2.54
	12.41	20.68		2.56	2.11	4.67	3.85	2.12	5.97
	13.41	16.89		3.80	1.35	5.15	0.62	1.31	1.93
	12.70	13.64		2.56	1.87	4.43	1.34	1.78	3.12
	11.41	28.30		3.12	1.25	4.37	3.39	3.86	7.25
	10.11	11.43		3.83	2.20	6.03			
	11.88	11.43		3.07	1.55	4.62			
	13.21	12.45		3.42	1.38	4.80			
	11.70	9.95		1.29	0.94	2.23			
	4.50	9.39		1.22	1.22	2.44			
	5.94	8.76		0.90	0.78	1.68			
	3.30	11.88		0.79	0.25	1.04			
	6.18	10.42		0.36	0.97	1.33			
	8.53	8.07		1.33	0.81	2.14			
	6.25	6.13		1.01	0.76	1.77			
	5.40	5.88		1.33	0.52	1.85			
	6.70			2.59	1.32	3.91			
	4.20								
	5.88								

Fig. S7. Quantification of endodermal expression domains for Pdx1 and Ptf1a upon overexpression of Hnf1ß

Area size of Pdx1 and endodermal Ptf1a expression domains (orange dotted lines) were estimated by ImageJ and the ratio to the whole endoderm (green dotted line) was calculated. A series of control and Hnf1ß injected embryos from two independent experiments (A and B) is displayed.

Development • Supplementary information

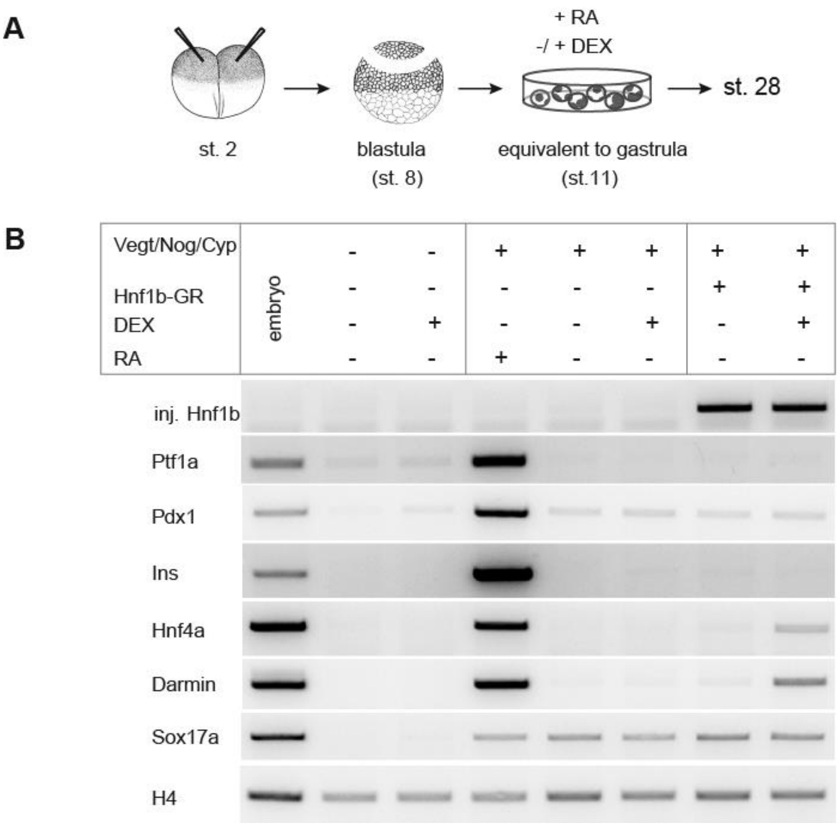

Fig. S8. Hnf1β is not a sufficient substitute for the induction of pancreatic gene expression by RA

(A) Programmed explants were from embryos co-injected with Hnf1β-GR RNA at the two-cell stage. Treatment with dexamethasone (DEX) and RA was at the equivalent of gastrula stage. At the equivalent of stage 28, total RNA was isolated and analyzed by RT-PCR. (B) RT-PCR analysis for pancreatic and endodermal genes as well as for the known direct Hnf1β target Hnf4α.

Development • Supplementary information

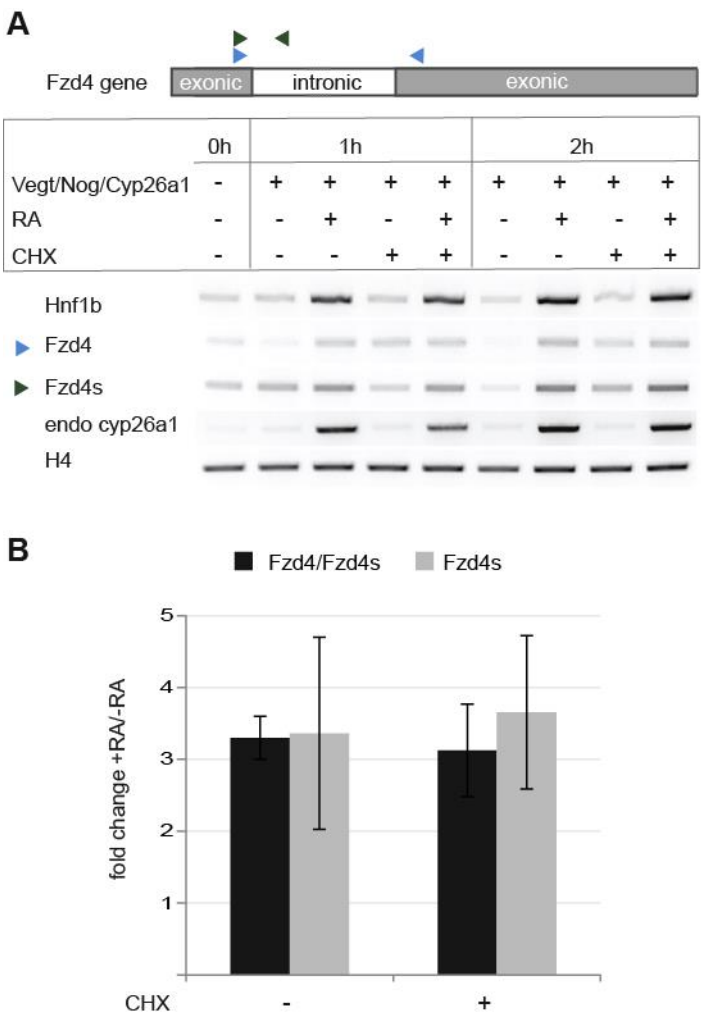

Fig. S9. Fzd4 and the splice variant Fzd4s are direct RA-target genes

(A) RT-PCR analysis with explants programmed as indicated making use of oligonucleotides distinguishing between Fzd4 and Fzd4s transcripts. (B) Transcript-specific determination of Fzd4/Fzd4s abundances. RNA sequencing reads mapping to the whole fzd4 gene region (Fzd4/Fzd4s) and the annotated fzd4 intron only (Fzd4s) were estimated. The fold change of transcript numbers in the absence or presence of RA is shown.

Development • Supplementary information

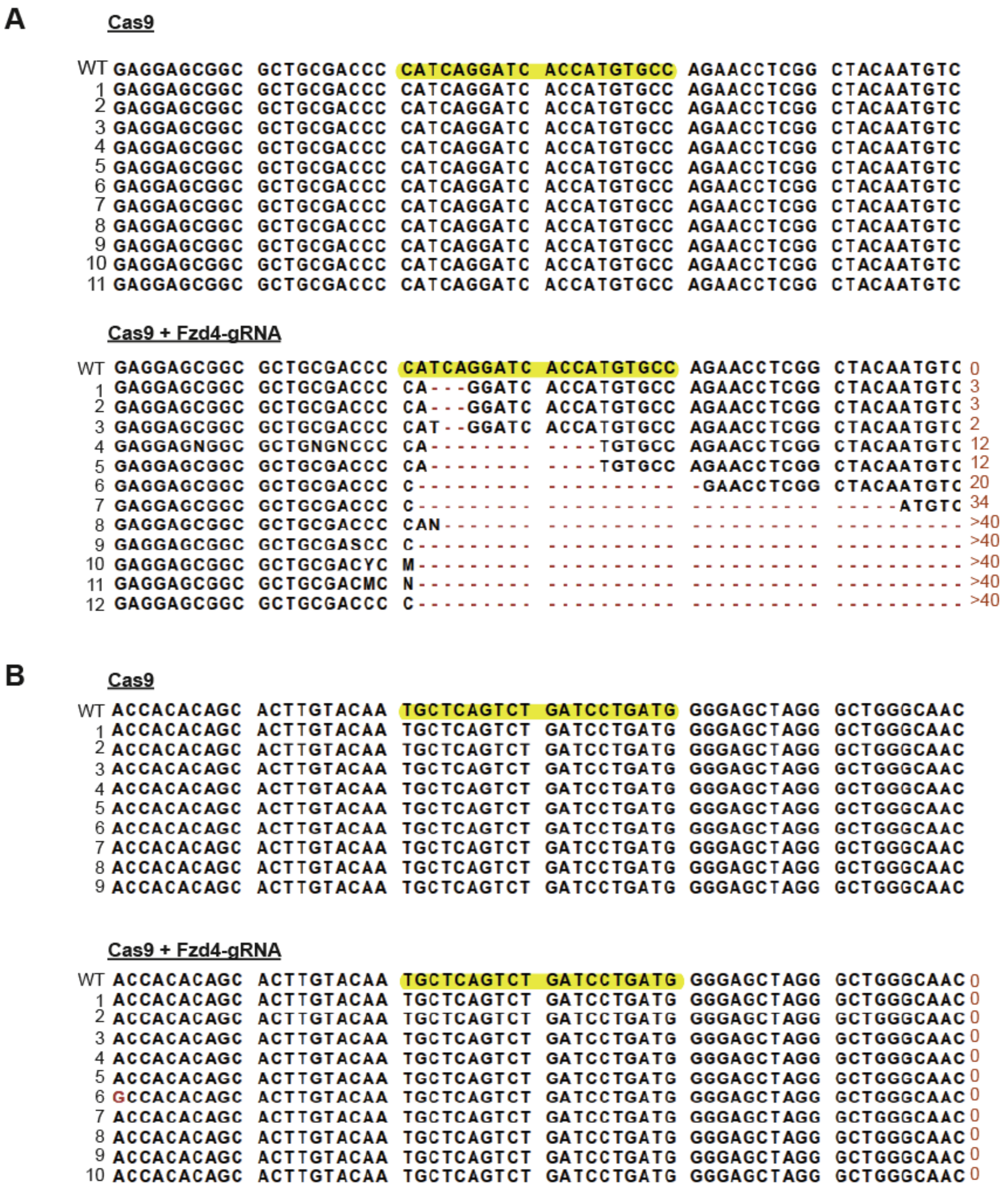

Fig. S10. Mutation analysis for the genomic locus of Fzd4 exon1 and the putative off-target Kremen2 in CRISPR/Cas-injected pancreatic explants

DNA sequences of (A) Fzd4 and (B) Kremen2 amplicons from CRISPR/Cas injected pancreatic explants were aligned to genomic *X.leavis* Fzd4 and Kremen2 sequences. The Fzd4-gRNA target sequence is highlighted in yellow and the number of deleted nucleotides indicated.

Development • Supplementary information

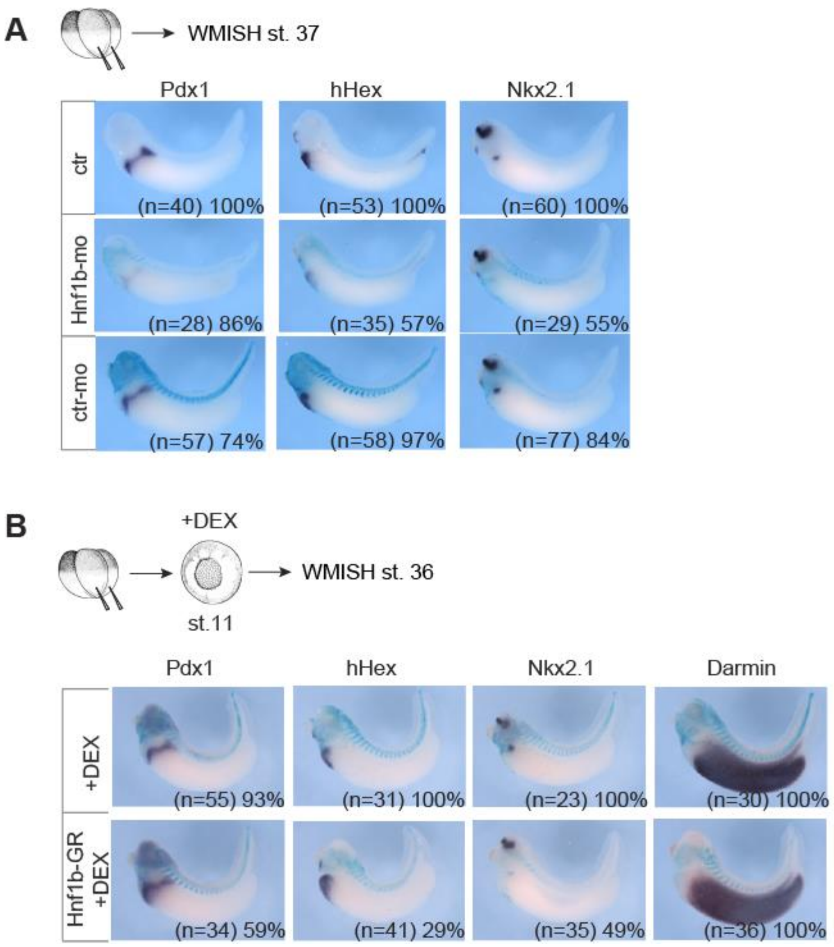

Fig. S11. Effects of Hnf1β down- or upregulation on the expression of various endodermal organ marker genes

Four-cell stage embryos were injected with RNA encoding β-galactosidase and either (A) Hnf1β-morpholino or control-morpholino, or (B) RNA coding for Hnf1β-GR, vegetally into the two dorsal blastomeres. At stage 36/37 embryos were used for WMISH against Pdx1 (pancreas, duodenum), hHex (liver), Nkx2.1 (thyroid, lung) and (B) Darmin (posterior endoderm).

Development • Supplementary information

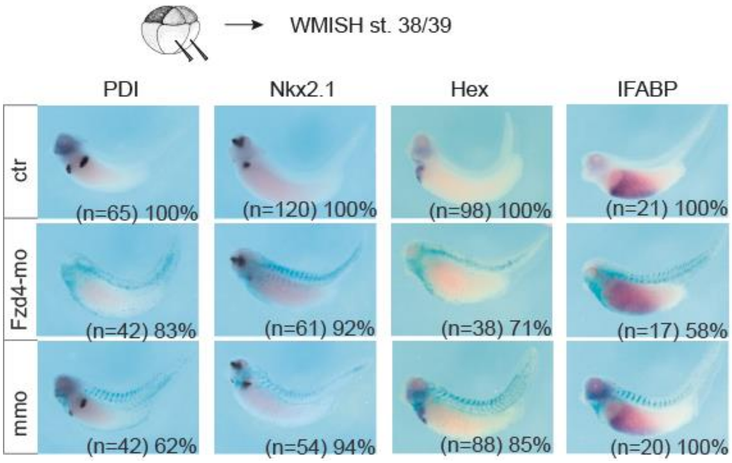

Fig. S12 Effects of Fzd4/Fzd4s downregulation on the expression of various endodermal organ marker genes

Eight-cell stage embryos were injected with RNA coding for β-galactosidase and either Fzd4/ Fzd4s-morpholino or the corresponding mismatch-morpholino. At stage 38/39, embryos were used for WMISH against Pdx1 (pancreas, duodenum), hHex (liver), Nkx2.1 (thyroid, lung) and IFABP (stomach, intestine).

Development • Supplementary information

Table S1. Nanostring analysis with embryos and pancreatic explants

	stage	17				24				32				39				43			
**tissue**	**gene\sample**	**CE**	**ctr**	**VNRA**	**VNC**	**CE**	**ctr**	**VNRA**	**VNC**	**CE**	**ctr**	**VNRA**	**VNC**	**CE**	**ctr**	**VNRA**	**VNC**	**CE**	**ctr**	**VNRA**	**VNC**
**Endoderm**	darmin	4785	1	6032	1	20699	1	17162	459	32301	2	14519	1747	21567	1	8050	265	4205	2	307	230
	sox17a-a	716	1	362	925	529	8	28	141	88	6	104	50	79	1	158	37	75	1	170	46
	sox17b	375	1	20	119	115	1	1	1	3	1	1	1	1	1	3	1	1	1	1	1
**Mesoderm**	*t-a/b*	1208	22	517	1164	392	88	260	300	118	85	157	193	49	34	110	98	24	72	80	101
	chrd	3489	25	9326	22713	1251	21	1629	2483	260	33	627	1007	161	35	249	363	57	21	47	123
	ventx2.1-b	1386	5843	208	1526	1045	1699	72	237	290	325	5	129	49	4	7	55	1	11	2	104
**Neuronal Ectoderm**	sox1	84	40	23	35	177	119	48	27	163	194	54	160	135	96	29	180	106	90	16	61
	sox2	1935	169	1939	2836	2843	243	1172	2038	2296	196	2025	3688	2652	173	2411	5225	2885	640	1956	4104
	sox3	2582	715	976	1899	2297	178	248	770	1254	169	453	986	1130	146	184	812	842	322	67	488
**Pancreas Progenitor**	ptf1a-a/b	19	32	14	14	25	67	340	6	190	127	2257	37	288	60	3581	1	279	109	2210	11
	pdx1	1	1	2	1	1	1	705	1	65	8	2549	5	117	1	2331	1	113	4	960	1
**Pancreas Differentiation**	ins-a	1	2	1	1	2	2	1886	1	51	4	14459	3	238	1	36320	1	895	1	29702	1
	ins-b	1	12	1	4	6	11	1028	1	52	20	10442	5	185	9	29422	1	918	18	22248	1
	pdia	65	194	67	142	167	649	136	98	151	1010	273	127	271	753	4018	120	2132	757	21802	130
	amy2a/b	45	153	98	140	95	496	111	76	84	650	80	92	93	309	106	69	381	370	4300	99
	tm4sf3	1	6	10	7	16	35	4	2	13	71	1	1	21	14	82	1	1807	37	3405	4
**Lung Differentiation**	sftpc	1	36	36	18	17	122	12	7	11	100	12	9	99	88	4	1	6262	126	23	34
**RA signaling**	cyp26a1	985	159	3038	633	852	311	832	742	955	337	889	604	799	57	207	169	694	151	58	101
	aldh1a2 (raldh2)	2088	13	270	1161	769	12	63	131	482	40	454	108	982	39	719	265	2103	131	1028	1124
	crabp2	5599	36	5065	13005	3273	128	2968	7701	2154	136	2988	4389	2264	65	3064	5215	1452	307	2038	5064
**Housekeeping**	odc	27372	27377	27375	27377	27365	27377	27371	27370	27353	27365	27361	27378	27364	27365	27350	27346	27342	27371	27366	27366
	actb	8.E+04	2.E+05	9.E+04	1.E+05	2.E+05	7.E+05	2.E+05	1.E+05	2.E+05	1.E+06	2.E+05	1.E+05	2.E+05	7.E+05	1.E+05	1.E+05	2.E+05	6.E+05	2.E+05	2.E+05
	g6pd	266	360	190	305	405	408	341	248	535	803	385	307	867	943	515	369	1551	3238	1194	977
	gapdh	485	169	5928	3112	2568	514	14505	8464	5916	4905	26893	17797	10729	22256	40000	26378	10120	41337	24386	22134

Normalized Nanostring counts are shown as mean values from two independent experiments. CE=whole embryo, ctr=unprogrammed explant, VNRA=pancreatic explants programmed with with VegT/Noggin/RA, VNC=VegT/Nog-programmed explant with co-injected CYP26a1

Development • Supplementary information

Table S2. RNA-sequencing data two hours after RA-addition in the absence of CHX.

no.	symbol	accession	gene function	UnigeneID_XL	log2FC +RA/-RA	FDR	- RA A	- RA B	+ RA A	+ RA B
1	*mesp2*				4.80	0.11%	1	1	138	10
2	*hoxa1*	BC080895,NM_001008016	homeodomain transcription factor	Xl.23512,Xl.283	4.72	0.00%	70	24	1295	1129
3	*hoxa2*	CU025162	homeodomain transcription factor	Xl.12098,Xl.751	4.45	0.00%	18	9	334	276
4	*Xl.8753*			Xl.8753	4.02	0.00%	2	4	64	65
5	*mespa*	CR848619,NM_001045719, BC157312	transcription factor	Xl.55,Xl.54	3.96	0.17%	89	96	3066	248
6	*hoxa3*	NM_001127429,BC166398	homeodomain transcription factor	Xl.9439	3.71	0.00%	18	6	153	173
7	*Xl.15091*			Xl.15091	3.65	0.00%	11	16	224	157
8	*dusp6*	NM_001045578,CR848381, BC118778,BC135306	MAP kinase phosphatase	Xl.31935,Xl.49155,Xl.52935,Xl.80026	3.09	0.00%	619	512	6084	3726
9	*znf703*	NM_001030507,BC093467	Zn-finger	Xl.1529,Xl.23634,Xl.69973	2.95	0.00%	1013	263	4168	4062
10	*Xl.9874*			Xl.9874	2.87	0.00%	18	35	160	240
11	*hoxa5*	BC088772,NM_001011405	homeodomain transcription factor	Xl.58357	2.86	0.00%	29	35	226	252
12	*dhrs3*	NM_001008431,CR848157, BC080136,CR761999		Xl.77868,Xl.59231,Xl.83090	2.85	0.00%	1015	1453	8872	8548
13	*cebpd*	NM_001030414,CR942782, BC091029	basic-leucine zipper (bZIP) transcription factor	Xl.29876,Xl.25857,Xl.81500	2.82	0.00%	38	24	158	238
14	*s1pr5*	BC161459,NM_001127068		Xl.75787,Xl.9971	2.75	0.03%	82	43	390	481
15	*hoxb4*	BC090114,BC161550,NM_001123015	Transcription factor zerknullt and related HOX domain proteins	Xl.54392,Xl.49715,Xl.72333	2.70	0.00%	51	14	192	200
16	*cyp26a1*	NM_001016147,CR761993, BC171087,BC167271	retinoic acid hydroxylase	Xl.50113,Xl.456	2.65	0.00%	3148	3665	18022	26048
17	*nodal1*	BC171037,CR761456,NM_001016321	transforming growth factor beta and bone morphogenetic	Xl.1037,Xl.85704	2.55	0.04%	21	35	240	75

Development • Supplementary information

no.	symbol	accession	gene function	UnigeneID_XL	log2FC +RA/-RA	FDR	- RA		+ RA	
							A	B	A	B
			protein related protein							
18	*Xl.67202*			Xl.67202	2.49	0.04%	37	22	154	193
19	*hnf1b*	CR760425,XM_002939634, XM_002939633	homeodomain transcription factor	Xl.65135,Xl.12667	2.47	0.00%	518	518	2808	2942
20	*hoxd1*	CR760220,NM_001016678, BC170965,BC170961,BC160395	homeodomain transcription factor	Xl.53491,Xl.11612,Xl.76294	2.42	0.00%	1020	306	3243	3015
21	*sox9*	NM_001016853,CR855424	HMG-box transcription factor	Xl.28992,Xl.1690,Xl.82068	2.36	0.00%	51	29	157	270
22	*Xl.82687*			Xl.82687	2.35	0.00%	12	13	39	103
23	*bhlhe40*	BC168818,BC169139,XM_002938312,BC154864,BC122074	basic helix-loop-helix (bHLH) transcription factor, circadian regulator	Xl.19778,Xl.26256	2.31	0.00%	79	76	399	375
24	*prph*	NM_001001235,BC067967	intermediate filament	Xl.27,Xl.16232	2.23	4.01%	9	7	11	93
25	*nkx6-2*	CT010540,XM_002937790	homeodomain transcription factor	Xl.55860,Xl.49827	2.13	0.00%	111	203	419	1028
26	*Xl.45046*			Xl.45046	2.13	0.00%	82	42	268	267
27	*rgs2*	BC091089,NM_001030451		Xl.49812,Xl.76046,Xl.85582	2.11	0.00%	52	26	234	111
28	*mespb*	NM_001016653,CR760340	transcription factor	Xl.51050,Xl.81675	2.08	4.27%	583	240	4138	453
29	*nr2f5*	XM_002938463	hormone receptor	Xl.1157,Xl.13423,Xl.81917,Xl.83500	2.07	0.00%	28	28	149	97
30	*neurog2*	XM_002934243	basic helix-loop-helix transcription factor	Xl.369,Xl.370,Xl.79613	2.05	0.04%	35	8	102	62
31	*trib1*	XM_002938661		Xl.75411,Xl.81850	2.03	0.88%	13	16	46	83
32	*Xl.84363*			Xl.84363	1.94	0.00%	37	38	158	137
33	*fst*	NM_001008056,BC080943	activin and bmp7 antagonist, also antagonizes bmp2/4 but less effectively	Xl.1094,Xl.75880,Xl.79342,Xl.83742	1.94	3.02%	131	974	1323	1718
34	*Xl.58101*			Xl.58101	1.88	0.00%	98	170	508	462

Development • Supplementary information

no.	symbol	accession	gene function	UnigeneID_XL	log2FC +RA/-RA	FDR	- RA		+ RA	
							A	B	A	B
35	*c10orf140*	NM_001126649,BC159015, BC159046		Xl.52870,Xl.80054	1.84	0.00%	119	59	294	340
36	*cxcr7*	NM_001030434,BC091057	glycoprotein hormone receptor	Xl.15037	1.84	0.01%	216	466	1409	817
37	*Xetrov72011149*				1.83	0.00%	28	29	94	111
38	*Xl.51509*			Xl.51509	1.82	0.11%	35	17	90	87
39	*Xl.6091*			Xl.6091	1.80	0.00%	32	21	109	81
40	*Xl.4906*			Xl.4906	1.63	0.00%	289	329	891	1022
41	*hunk*	NM_001127077,BC161479		Xl.24322,Xl.12483,Xl.830 64	1.61	0.00%	449	355	1193	1254
42	*tmem72*	BC158380,XM_002939555		Xl.17444	1.59	0.10%	38	24	97	87
43	*Xl.16263*			Xl.16263	1.51	0.00%	146	92	403	274
44	*txnip*	XM_002938464,BC121658		Xl.77253,Xl.13359,Xl.573 71,Xl.76363,Xl.78726	1.50	0.00%	123	111	470	224
45	*nodal2*	XM_002932721	transforming growth factor beta and bone morphogenetic protein related protein	Xl.1038	1.49	0.33%	88	279	506	412
46	*foxh1*	CR761447,NM_001017084	winged helix transcription factor	Xl.381	1.47	0.68%	70	66	183	200
47	*kiaa0182*	XM_002936097,BC125792		Xl.78028,Xl.1433,Xl.7827 6	1.47	0.00%	290	169	553	706
48	*meis3*	CR760178,BC075589,NM_ 001006781	homeodomain transcription factor	Xl.23066,Xl.452,Xl.80020, Xl.82977	1.46	0.00%	2458	1457	5909	4590
49	*Xl.71159*			Xl.71159	1.45	0.00%	160	153	556	316
50	*znf503*	BC124040,NM_001079230	zinc finger, C2H2 type	Xl.60819,Xl.80579	1.44	0.00%	437	380	926	1324
51	*myc*	BC064880,NM_204059,CR 761143		Xl.826,Xl.1155	1.43	0.00%	1447	527	2915	1943
52	*igf3*	BC161167,CR848377,NM_ 001126946		Xl.12078,Xl.78527	1.43	0.00%	49	35	122	107
53	*fgf16*	XM_002931813	heparin-binding growth factor		1.42	0.00%	289	322	893	746
54	*Xl.74263*			Xl.74263	1.42	0.01%	153	82	262	339

Development • Supplementary information

no.	symbol	accession	gene function	UnigeneID_XL	log2FC +RA/-RA	FDR	- RA		+ RA	
							A	B	A	B
55	*tdgf1p2*	XM_002940604	EGF-like growth factor	Xl.15503,Xl.51406	1.41	0.10%	32	42	112	88
56	*gbx2.1*	XM_002932000,XM_002932001	homeodomain transcription factor	Xl.77345	1.41	0.08%	74	53	169	166
57	*cdx4*	AF417199,CT030483,BC167866,NM_204086,BC170869,BC170865	homeodomain transcription factor	Xl.26888	1.39	0.03%	2515	1094	6302	3014
58	*Xl.57027*			Xl.57027	1.38	0.10%	29	34	78	88
59	*Xl.80298*			Xl.80298	1.38	0.21%	47	32	106	99
60	*Xetrov72036339*				1.36	0.00%	108	135	213	440
61	*Xetrov72039973*				1.35	3.33%	27	31	63	88
62	*gadd45a*	NM_001016151,BC167898,BC121532,CR761969	40S ribosomal protein S12	Xl.79095,Xl.71159,Xl.81981	1.33	0.22%	1216	1180	4394	1907
63	*stox1*	XM_002936869	Conserved protein Knockout	,Xl.15086	1.32	0.02%	1164	668	1955	2459
64	*fzd4*	XM_002936543	transmembrane receptor in the wnt signalling pathway	Xl.460,Xl.53888	1.32	0.00%	681	503	1365	1581
65	*hoxa4*		homeodomain transcription factor		1.32	0.72%	163	85	318	286
66	*Xl.68408*			Xl.68408	1.31	1.33%	35	45	59	151
67	*dusp5*	BC090366,NM_001015856	MAP kinase phosphatase	Xl.79939,Xl.15374	1.26	0.11%	799	620	1677	1690
68	*Xl.80297*			Xl.80297	1.26	0.45%	213	316	813	440
69	*tril*	XM_002933390		Xl.73400,Xl.78580	1.26	0.00%	821	401	1596	1160
70	*Xl.57926*			Xl.57926	1.24	0.03%	867	751	2007	1811
71	*erf*	NM_001015821,BC090103	transcription factor	Xl.41820,Xl.47734,Xl.78801	1.22	2.07%	691	307	961	1174
72	*Xl.70850*			Xl.70850	1.21	0.00%	81	121	176	298
73	*lhx1*	NM_001100228,BC135731	LIM and homeodomain containing transcription factor	Xl.32655,Xl.79655,Xl.81258,Xl.82262	1.19	0.18%	2515	4358	8329	6707
74	*hoxd4*	XM_002935672,XM_002935673	homeodomain transcription factor	Xl.34346,Xl.85858	1.17	4.14%	60	65	91	207

Development • Supplementary information

no.	symbol	accession	gene function	UnigeneID_XL	log2FC +RA/-RA	FDR	- RA		+ RA	
							A	B	A	B
75	*fhdc1*	CR942785,XM_002933493			1.17	0.07%	897	632	1490	1885
76	*rgs14*	XM_002938062		Xl.14836	1.15	0.64%	48	54	88	142
77	*kirrel2*	NM_001142126,BC168130	Immunoglobulin C-2 Type/fibronectin type III domains	Xl.76492,Xl.60720,Xl.76486,Xl.81573	1.11	1.50%	541	706	1333	1350
78	*pim1*	NM_001008131,BC081340,CR760428		Xl.48564,Xl.68517,Xl.78542	1.11	0.30%	1575	1405	4311	2312
79	*dact1*	BC080457,CR761983,NM_001007949	antagonist of beta-catenin and c-jun N-terminal kinase signalling	Xl.7602,Xl.19185	1.11	0.05%	3586	2838	8553	5424
80	*Xl.47239*			Xl.47239	1.10	0.07%	126	96	264	211
81	*rara*	NM_001171194,CT025407	transcription factor, steroid receptor class	Xl.158,Xl.57139	1.09	1.03%	250	162	387	472
82	*pkdcc.1*	BC168090,NM_001142110		Xl.80884,Xl.55752,Xl.59589	1.07	0.98%	774	1264	2368	1864
83	*mxi1*	NM_001008128,CR942348,BC081336	transcription factor	Xl.50498	1.05	1.27%	286	218	398	635
84	*cass4*	XM_002942273	p53-interacting protein 53BP/ASPP, contains ankyrin and SH3 domains	Xl.22909	1.04	0.51%	258	324	507	700
85	*Xl.79790*			Xl.79790	1.04	2.54%	29	38	41	103
86	*Xl.82247*			Xl.82247	1.03	0.62%	213	190	459	371
87	*fgfr4*	CR761450,NM_001016323,BC170953,BC170565,BC161582	receptor tyrosine kinase	Xl.1008,Xl.1016	1.02	0.00%	2621	2113	5280	4345
88	*slc38a8*	CR760593,NM_001044435		Xl.70342,Xl.78242	1.02	1.11%	550	803	1420	1269
89	*hey1*	CR760684,NM_001007910,BC080349	helix-loop-helix transcription factor/transcriptional repressor	Xl.469,Xl.7544,Xl.76323	1.02	0.02%	185	162	396	311
90	*hoxb3*	CR848404,BC135406,NM_001015971	homeodomain transcription factor	Xl.79878,Xl.80826	1.02	0.09%	117	92	193	230
91	*mn1*	NM_001100202,BC135419	Chitinase	Xl.57334,Xl.69093,Xl.828	1.01	0.98%	598	499	1198	1017

no.	symbol	accession	gene function	UnigeneID_XL	log2FC +RA/-RA	FDR	- RA		+ RA	
							A	B	A	B
				18						
92	*spry2*	AY714335,BC064204,NM_001006931,CR761255	regulation of signal transduction	Xl.11964,Xl.11965	1.01	0.99%	1781	1675	3743	3226
93	*Xl.85251*			Xl.85251	-1.06	0.75%	85	80	43	35
94	*Xl.13572*			Xl.13572	-1.06	0.47%	130	112	56	59
95	*twist1*	NM_204084,BK006266,BC074558,CR760828,AF176819	transcription factor	Xl.879,Xl.23366,Xl.68451,Xl.82751,Xl.84873	-1.17	1.23%	217	260	101	108
96	*pdgfb*	XM_002933750	growth factor	Xl.14185,Xl.13630	-1.59	0.00%	218	252	102	61

Normalized number of mapped reads in the presence and absence of RA for two independent experiments (A and B). Genes marked with an asterisk (∗) could not been analyzed by Nanostring.

Development • Supplementary information

Table S3. RNA-sequencing data two hours after RA-addition in the presence of CHX.

no.	symbol	accession	gene function	UnigeneID_XL	log2FC +RA/-RA	FDR	-RA +CHX		+RA +CHX	
							A	B	A	B
1	*hoxa1*	BC080895,NM_001008016	homeodomain transcription factor	Xl.23512,Xl.283	4.82	0.00%	53	52	1352	1639
2	*hoxa3*	NM_001127429,BC166398	homeodomain transcription factor	Xl.9439	4.64	0.00%	9	7	167	286
3	*hoxa2*	CU025162	homeodomain transcription factor	Xl.12098,Xl.751	3.82	0.00%	21	21	314	304
4	*hoxb4*	BC090114,BC161550,NM_001123015	Transcription factor zerknullt and related HOX domain proteins	Xl.54392,Xl.49715,Xl.72333	3.68	0.00%	27	14	216	286
5	*foxi4.2*	BC158378,NM_203934,BC064241,CR761756	forkhead domain transcription factor	Xl.5369,Xl.34912	3.42	4.51%	140	14	658	512
6	*hoxb1*	XM_002938017	homeodomain transcription factor	Xl.85422	3.27	0.00%	11	12	130	102
7	*Xl.78389*			Xl.78389	3.25	4.90%	61	10	271	245
8	*Xl.15091*			Xl.15091	3.07	0.00%	13	5	84	76
9	*hoxa5*	BC088772,NM_001011405	homeodomain transcription factor	Xl.58357	3.02	0.00%	29	43	209	403
10	*Xl.67202*			Xl.67202	2.72	0.01%	26	58	220	338
11	*cyp26c1*	XM_002939091	electron transport	Xl.82105,Xl.1946,Xl.41946	2.72	0.00%	6	9	51	58
12	*znf703*	NM_001030507,BC093467	Zn-finger	Xl.1529,Xl.23634,Xl.69973	2.71	0.01%	1404	778	5731	6984
13	*cyp26a1*	NM_001016147,CR761993,BC171087,BC167271	retinoic acid hydroxylase	Xl.50113,Xl.456	2.50	0.00%	2167	5301	16230	22874
14	*s1pr5*	BC161459,NM_001127068		Xl.75787,Xl.9971	2.48	0.25%	83	129	532	657
15	*Xl.82687*			Xl.82687	2.47	0.00%	9	9	28	91
16	*sox9*	NM_001016853,CR855424	HMG-box transcription factor	Xl.28992,Xl.1690,Xl.82068	2.34	0.00%	39	70	179	382
17	*dusp6*	NM_001045578,CR848381,BC118778,BC135306	MAP kinase phosphatase	Xl.31935,Xl.49155,Xl.52935,Xl.80026	2.30	0.00%	1363	1003	5859	5629
18	*Xl.45046*			Xl.45046	2.25	0.00%	80	51	279	334

Development • Supplementary information

no.	symbol	accession	gene function	UnigeneID_XL	log2FC +RA/-RA	FDR	-RA +CHX		+RA +CHX	
							A	B	A	B
19	*dhrs3*	NM_001008431,CR848157,BC080136,CR761999		Xl.77868,Xl.59231,Xl.83090	2.24	0.00%	1510	1943	7370	8933
20	*trib1*	XM_002938661		Xl.75411,Xl.81850	2.23	0.15%	32	14	77	154
21	*hoxd1*	CR760220,NM_001016678,BC170965,BC170961,BC160395	homeodomain transcription factor	Xl.53491,Xl.11612,Xl.76294	2.22	0.01%	744	614	3612	2775
22	*Xl.9874*			Xl.9874	2.20	0.00%	31	55	154	243
23	*cebpd*	NM_001030414,CR942782,BC091029	basic-leucine zipper (bZIP) transcription factor	Xl.29876,Xl.25857,Xl.81500	2.16	0.63%	122	27	332	252
24	*bhlhe40*	BC168818,BC169139,XM_002938312,BC154864,BC122074	basic helix-loop-helix (bHLH) transcription factor, circadian regulator	Xl.19778,Xl.26256	2.08	0.00%	129	71	508	337
25	*hnf1b*	CR760425,XM_002939634,XM_002939633	homeodomain transcription factor	Xl.65135,Xl.12667	2.08	0.00%	636	480	2760	1958
26	*gbx2.2*	BC088605,NM_001011472	homeodomain transcription factor	Xl.973	2.02	0.96%	12	17	60	65
27	*hoxc4*	XM_002936638	homeodomain transcription factor	Xl.69442	1.97	0.25%	29	21	89	110
28	*Xl.51509*			Xl.51509	1.97	0.07%	31	13	94	74
29	*tmem72*	BC158380,XM_002939555		Xl.17444	1.96	0.01%	25	9	102	39
30	*gata3*	NM_001004967,BC075479	zinc finger transcription factor	Xl.28002	1.95	4.90%	186	73	389	471
31	*neurog2*	XM_002934243	basic helix-loop-helix transcription factor	Xl.369,Xl.370,Xl.79613	1.83	0.18%	60	23	156	119
32	*c10orf140*	NM_001126649,BC159015,BC159046		Xl.52870,Xl.80054	1.81	0.00%	126	128	355	532
33	*gbx2.1*	XM_002932000,XM_002932001	homeodomain transcription factor	Xl.77345	1.79	0.00%	75	75	253	272
34	*nkx6-2*	CT010540,XM_002937790	homeodomain transcription factor	Xl.55860,Xl.49827	1.71	0.00%	249	281	580	1333
35	*Xl.29118*			Xl.29118	1.70	1.64%	11	36	63	83
36	*rgs2*	BC091089,NM_001030451		Xl.49812,Xl.76046,Xl.85582	1.69	0.00%	88	28	249	119
37	*foxh1*	CR761447,NM_001017084	winged helix transcription factor	Xl.381	1.62	0.17%	85	80	201	316

Development • Supplementary information

no.	symbol	accession	gene function	UnigeneID_XL	log2FC +RA/-RA	FDR	-RA +CHX		+RA +CHX	
							A	B	A	B
38	*Xl.58101*			Xl.58101	1.59	0.00%	101	109	283	358
39	*Xl.6091*			Xl.6091	1.55	0.01%	35	26	90	91
40	*klb*	XM_002933470	beta-glucosidase		1.52	0.00%	31	24	79	81
41	*hoxd4*	XM_002935672,XM_002935673	homeodomain transcription factor	Xl.34346,Xl.85858	1.49	0.19%	47	51	128	150
42	*olig4*	BC161514,NM_001045715,CR848409	transcription factor	Xl.72230	1.46	0.00%	45	35	113	109
43	*hoxb8*	XM_002938021	homeodomain transcription factor	Xl.79158,Xl.9701	1.42	0.13%	39	17	105	49
44	*Xl.84363*			Xl.84363	1.37	0.45%	67	58	146	178
45	*hoxb3*	CR848404,BC135406,NM_001015971	homeodomain transcription factor	Xl.79878,Xl.80826	1.35	0.00%	69	75	202	171
46	*hoxa4*		homeodomain transcription factor		1.29	1.70%	156	97	232	360
47	*Xl.13431*			Xl.13431	1.28	4.51%	23	17	61	41
48	*rara*	NM_001171194,CT025407	transcription factor, steroid receptor class	Xl.158,Xl.57139	1.21	0.24%	258	174	429	555
49	*myc*	BC064880,NM_204059,CR761143		Xl.826,Xl.1155	1.20	0.25%	1560	767	3167	1987
50	*igf3*	BC161167,CR848377,NM_001126946		Xl.12078,Xl.78527	1.20	0.18%	54	32	108	91
51	*nr2f5*	XM_002938463	hormone receptor	Xl.1157,Xl.13423,Xl.81917,Xl.83500	1.19	1.22%	46	43	136	74
52	*edn1*	XM_002932664,BC161190	secreted peptide precursor	Xl.68930,Xl.63796	1.16	0.57%	54	52	115	125
53	*hunk*	NM_001127077,BC161479		Xl.24322,Xl.12483,Xl.83064	1.15	0.03%	649	566	1215	1468
54	*Xl.79790*			Xl.79790	1.14	0.80%	22	49	55	102
55	*Xl.41047*			Xl.41047	1.11	0.56%	68	52	188	86
56	*meis3*	CR760178,BC075589,NM_001006781	homeodomain transcription factor	Xl.23066,Xl.452,Xl.80020,Xl.82977	1.09	0.07%	3434	2009	6277	5044
57	*kiaa0182*	XM_002936097,BC125792		Xl.78028,Xl.1433,Xl.78276	1.08	0.46%	329	297	615	709

no.	symbol	accession	gene function	UnigeneID_XL	log2FC +RA/-RA	FDR	-RA +CHX		+RA +CHX	
							A	B	A	B
58	*tril*	XM_002933390		Xl.73400,Xl.78580	1.05	0.16%	895	413	1695	941
59	*Xl.74263*			Xl.74263	1.04	4.90%	136	89	242	216
60	*fzd4*	XM_002936543	transmembrane receptor in the wnt signalling pathway	Xl.460,Xl.53888	0.93	0.69%	1032	741	1679	1690
61	*twist1*	NM_204084,BK006266,BC074558,CR760828,AF176819	transcription factor	Xl.879,Xl.23366,Xl.68451,Xl.82751,Xl.84873	-1.10	4.50%	261	222	82	155

Normalized number of mapped reads in the presence and absence of RA for two independent experiments (A and B).

Development • Supplementary information

Table S4. Verification of RA-responsiveness by Nanostring analysis

RA-induicibility				RA-dependency	
in vitro (52)		in vivo (48)		gene	fold change
gene	fold change	gene	fold change		
bhlhe40	2.05	*bhlhe40*	4.72	cebpd	2.05
cdx4	2.09	*cass4*	1.54	cyp26a1	1.81
cebpd	18.71	*cebpd*	3.35	dhrs3	1.78
cxcr7	1.69	*cyp26a1*	6.70	foxh1	1.72
cyp26a1	14.23	*dact1-a/b*	1.82	fst	1.91
dact1-a/b	1.51	*dhrs3*	4.13	fzd4	1.61
dhrs3	8.86	*dusp5*	1.96	gbx2.1	1.76
dusp5	2.28	*dusp6*	3.55	hnf1b	1.71
dusp6	5.39	*erf*	1.90	hoxa1-b	2.66
erf	1.50	*foxh1*	1.56	hoxb1	1.81
foxh1	2.49	*fst*	1.75	hoxd1	1.54
fst	1.73	*fzd4*	1.92	hoxd4	1.66
fzd4	2.26	*gbx2.1*	2.07	igf3	1.53
gbx2.1	4.56	*hnf1b*	2.47	lhx1	1.51
hey1	1.57	*hoxa1-a/b*	3.27	meis3	1.74
hnf1b	3.00	*hoxa2*	1.82	myc-a/b	1.56
hoxa1-a/b	44.33	*hoxa3*	108.71	neurog2	1.84
hoxb1	9.81	*hoxb1*	2.56	nkx6-2	2.39
hoxb1	21.42	*hoxb3*	25.56	prph	1.60
hoxb4	2.23	*hoxb4*	2.46	rara	1.60
hoxd1	2.54	*hoxd1*	1.75	twist1	1.52
hoxd4	2.33	*hoxd4*	6.07	Xl.45046	2.02
hunk	2.64	*hunk*	2.02	Xl.47239	1.77
igf3	3.38	*igf3*	1.92	Xl.51509	1.74
kiaa0182	2.04	*kiaa0182*	1.56	Xl.57926	1.53
kirrel2	1.87	*lhx1*	4.00	Xl.9822	2.29
lhx1	2.42	*meis3*	1.96	znf703	1.64
meis3	2.59	*mespa*	1.73		
mespa	7.19	*mxi1*	2.08		
mespb	3.53	*nkx6-2*	2.49		
mxi1	3.05	*nr2f5*	4.38		
myc	1.99	*pdgfb*	3.87		
nkx6-2	4.32	*pim1*	1.96		
nodal1	1.75	*prph*	4.68		
pim1	1.76	*rara*	1.89		
prph	31.75	*rgs2*	2.43		
rgs14	2.40	*sox9*	2.96		
rgs2	1.93	*spry2*	1.55		
sox9	1.60	*tmem72*	1.77		
spry2	1.61	*txnip*	2.10		
tmem72	2.74	*Xl.45046*	1.84		

RA-induicibility				RA-dependency	
in vitro (52)		in vivo (48)		gene	fold change
gene	fold change	gene	fold change		
trib1	4.58	*Xl.47239*	1.70		
txnip	7.40	*Xl.51509*	3.88		
Xl.45046	7.03	*Xl.57027*	2.72		
Xl.47239	3.34	*Xl.57926*	2.37		
Xl.4906	3.14	*Xl.79790*	2.18		
Xl.51509	1.63	*Xl.80297*	2.08		
Xl.57027	14.12	*znf703*	1.94		
Xl.57926	1.99				
Xl.79790	2.24				
Xl.80297	1.90				
Xl.9822	2.23				
znf703	8.98				

Numbers indicate the fold change of transcript number upon increased (inducibility) or decreased RA-signalling (dependency). See Fig. S3 for experimental details.

Development • Supplementary information

Table S5. Transcript-specific determination of Fzd4/Fzd4s abundances

variants	A				B			
	-CHX		+CHX		-CHX		+CHX	
	-RA	+RA	-RA	+RA	-RA	+RA	-RA	+RA
Fzd4/Fzd4s	761	2284	1242	3084	728	2618	843	3176
Fzd4s	74	150	70	181	33	155	25	118

Normalized mapped reads for Fzd4/Fzd4s non-discriminatory and Fzd4s specific transcripts for two independent experiments (A and B) obtained by RNA-sequencing.

Table S6. Predicted Fzd4-gRNA off-target sequences in exonic regions of the X. laevis genome

Coordinates	strand	MM	target_seq	PAM	gene name	gene id
Scaffold149581:4344665-4344687	+	4	GGAAGATG[GTGAACCTGAAG]	AGG	utp14a	XB-GENE-5954915
Scaffold230826:3712453-3712475	-	5	AGAGCAGG[TTGATCCTGATG]	AGG	impad1	XB-GENE-5751113
Scaffold102068:223547-223569	+	5	TGCTCAGT[CTGATCCTGATG]	GGG	kremen2	XB-GENE-866619
Scaffold139113:827475-827497	-	5	GTTTCATG[ATGGTCCTGATG]	CGG	fzd7	XB-GENE-483735
Scaffold30711:2325955-2325977	+	5	GCCTCCTG[ATGAGCCTGATG]	AGG	zmym4	XB-GENE-1217387
Scaffold7197:648108-648130	-	5	GGTACACC[GTTATTCTGATG]	TGG	edc3	XB-GENE-5890837
Scaffold47683:3850870-3850892	-	5	TATACAAG[GTGATCCTGGTG]	AGG	exog	XB-GENE-981842
Scaffold55171:287242-287264	+	5	GGGAATTG[GTGATCCTGTTA]	GGG	gcdh	XB-GENE-1016454

Highlighted in grey are the tested off-targets and indicated in red are the mismatches; PAM= protospacer adjacent motif; MM = mismatch

Development • Supplementary information
